# Supplementary material for: Stromal Cells Covering Omental Fat-Associated Lymphoid Clusters Trigger Formation of Neutrophil Aggregates to Capture Peritoneal Contaminants
Source: Immunity. 2020 Apr 14;52(4):700–715.e6. doi: 10.1016/j.immuni.2020.03.011 (PMC7156918; doi:10.1016/j.immuni.2020.03.011)
Supplement: Document S1. Figures S1–S7 and Tables S1 and S2 [file mmc1.pdf]

Immunity, Volume *52*

Supplemental Information

Stromal Cells Covering Omental Fat-Associated Lymphoid Clusters Trigger Formation of Neutrophil Aggregates to Capture Peritoneal Contaminants

Lucy Helen Jackson-Jones, Peter Smith, Jordan Raymond Portman, Marlène Sophie Magalhaes, Katie Jude Mylonas, Matthieu Marie Vermeren, Mark Nixon, Beth Emily Pollot Henderson, Ross Dobie, Sonja Vermeren, Laura Denby, Neil Cowan Henderson, Damian James Mole, and Cécile Bénézech

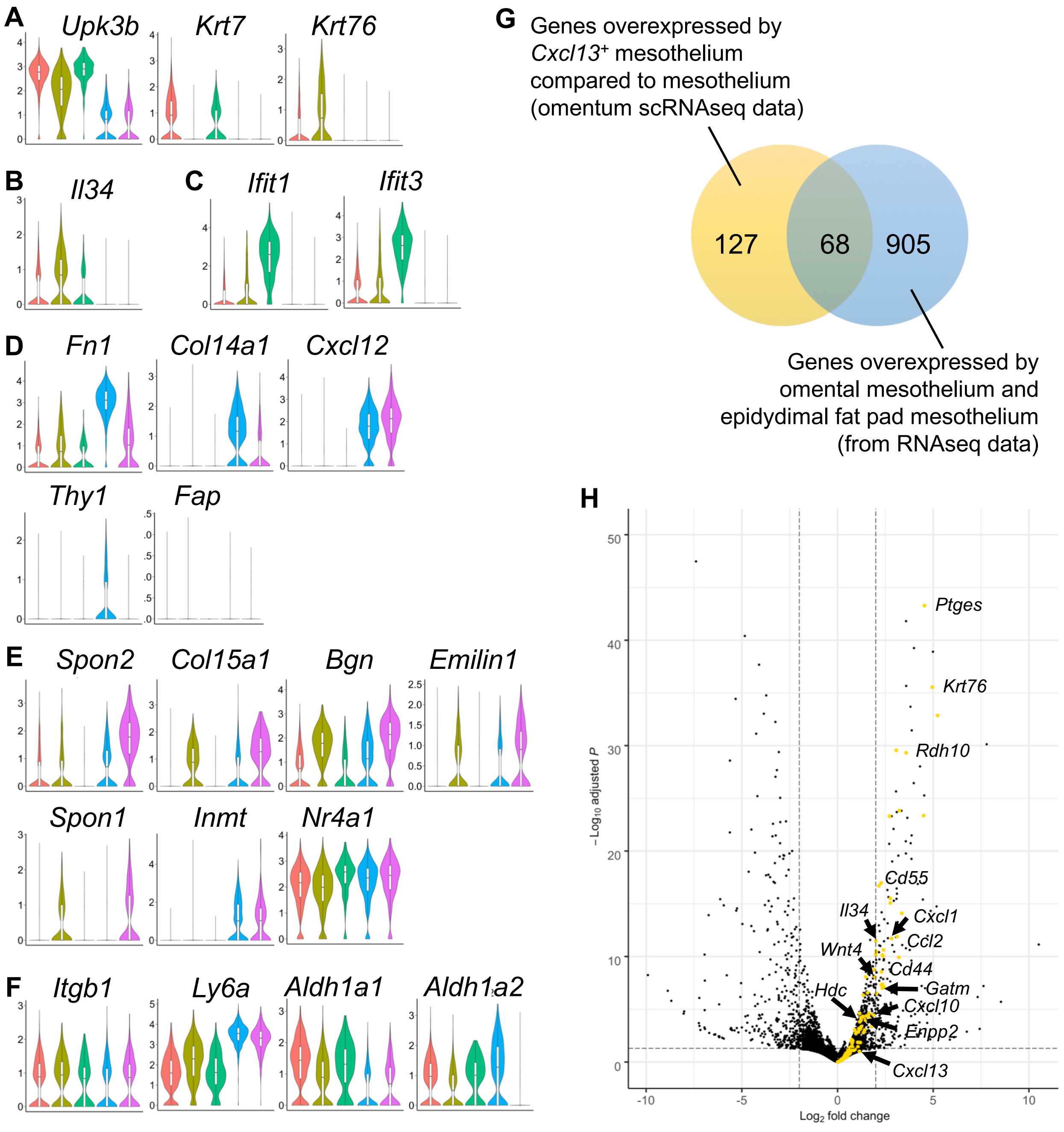

Figure S1. Related to Figure 1. (A-F) Violin plots of canonical omental stromal cell gene expression by cluster with highest log-normalized expression value labelled. Plots highlighting DEGs characteristic of mesothelial (**A, F**) immune (**B, C**) fibroblast (**D**) and ECM (**E**). **(G-H) Differential gene expression analysis between omentum mesothelial cells and epidydimal mesothelial cells. G,** Venn Diagram showing the number of genes significantly upregulated (adjusted p value <0.05) by the omental *Cxcl13*$^+$ mesothelial cell cluster compared to the omental mesothelial cell cluster (yellow circle, 195 total) and by *PDPN*$^+$*PDFGRa*$^-$ omental mesothelial cells compared to *PDPN*$^+$*PDFGRa*$^-$ epidydimal fat pad mesothelial cells (blue circle, 973 total) obtained from published RNAseq data GSM3754642, GSM3754643, GSM3754644, GSM3754627, GSM3754628, and GSM3754629) (Buechler et al., 2019). 68 DEG overlapping. **H**, Volcano plot showing DEG between omentum mesothelial cells and epididymal mesothelial cells in grey. Examples of genes that are also upregulated by the omental *Cxcl13*$^+$ mesothelial cell cluster compared to the omental mesothelial cell cluster are shown in yellow.

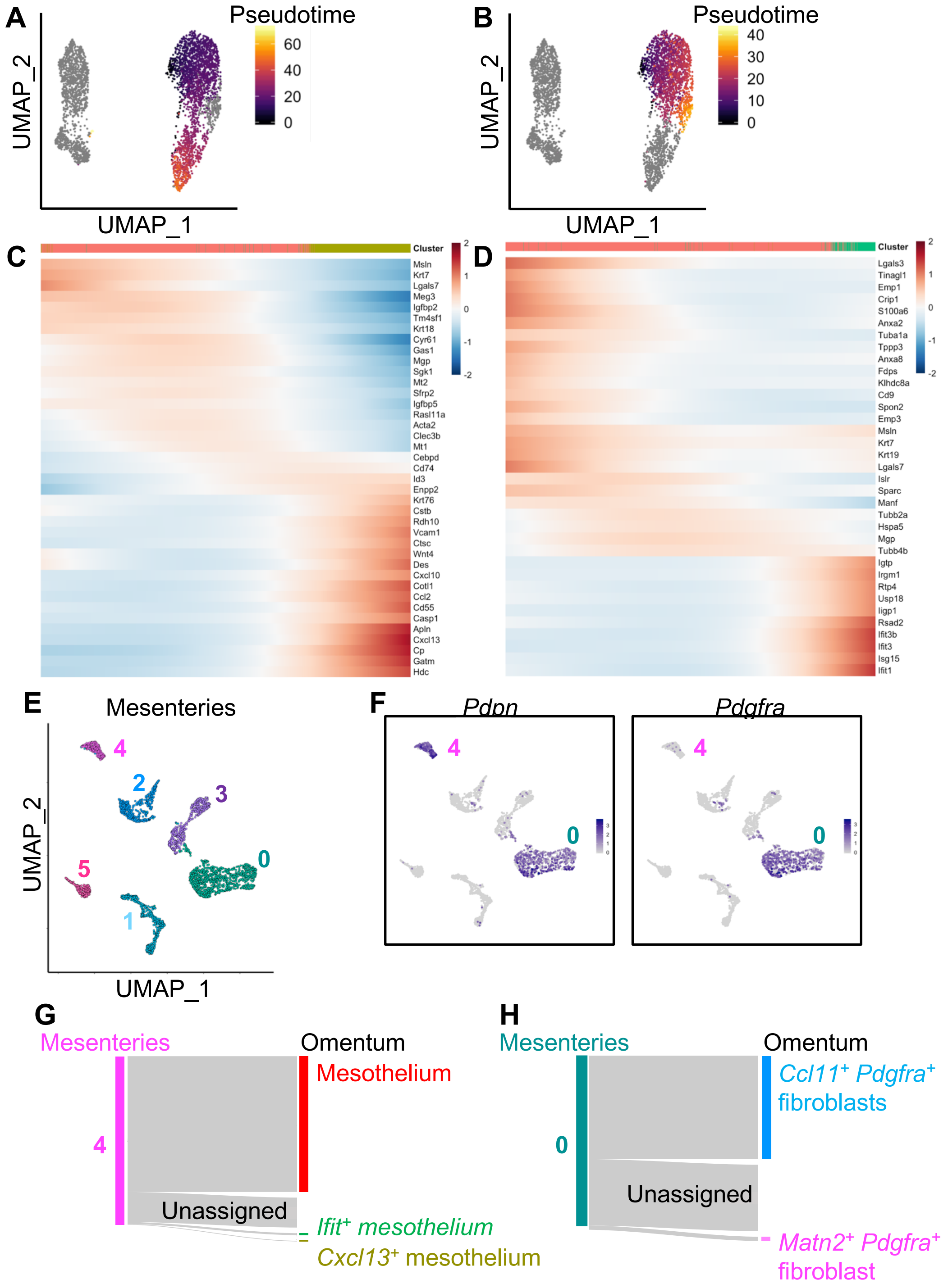

Figure S2. Mesothelium trajectory inference and mapping of mesenteric stromal cell subsets to omental stromal cell subsets related to Figure 1. A-B, UMAP visualization on (top panel) of the Pseudotime values from Mesothelium (starting point) to *Cxcl13*$^{+}$ mesothelium (**A**) and *Ifit*$^{+}$ mesothelium (**B**). **C-D**, Heat map with spline curves fitted to genes differentially expressed along a trajectory from mesothelium to *Cxcl13*$^{+}$ mesothelium (**C**) and *Ifit*$^{+}$ mesothelium (**D**). **E**, Unsupervised clustering of mesenteric cells visualized with UMAP from the published single cell dataset GSE102665 (Koga et al., 2018). **F,** Gene expression of *Pdpn* and *Pdgfra* distinguishing *Pdpn*$^{+}$*Pdgfra*$^{-}$ mesenteric mesothelial cells (cluster 4, pink) and *Pdpn*$^{+}$*Pdgfra*$^{+}$ mesenteric fibroblasts (cluster 0, dark green) projected onto UMAP plots. Color scaled for each gene with highest log-normalized expression level noted. **G-H**, Mapping of the *Pdgfra*$^{+}$*Pdpn*$^{-}$ mesenteric mesothelial cell cluster (**G**) and the *Pdgfra*$^{+}$*Pdpn*$^{+}$ mesenteric fibroblast cluster (**H**) to the omentum scRNAseq dataset.

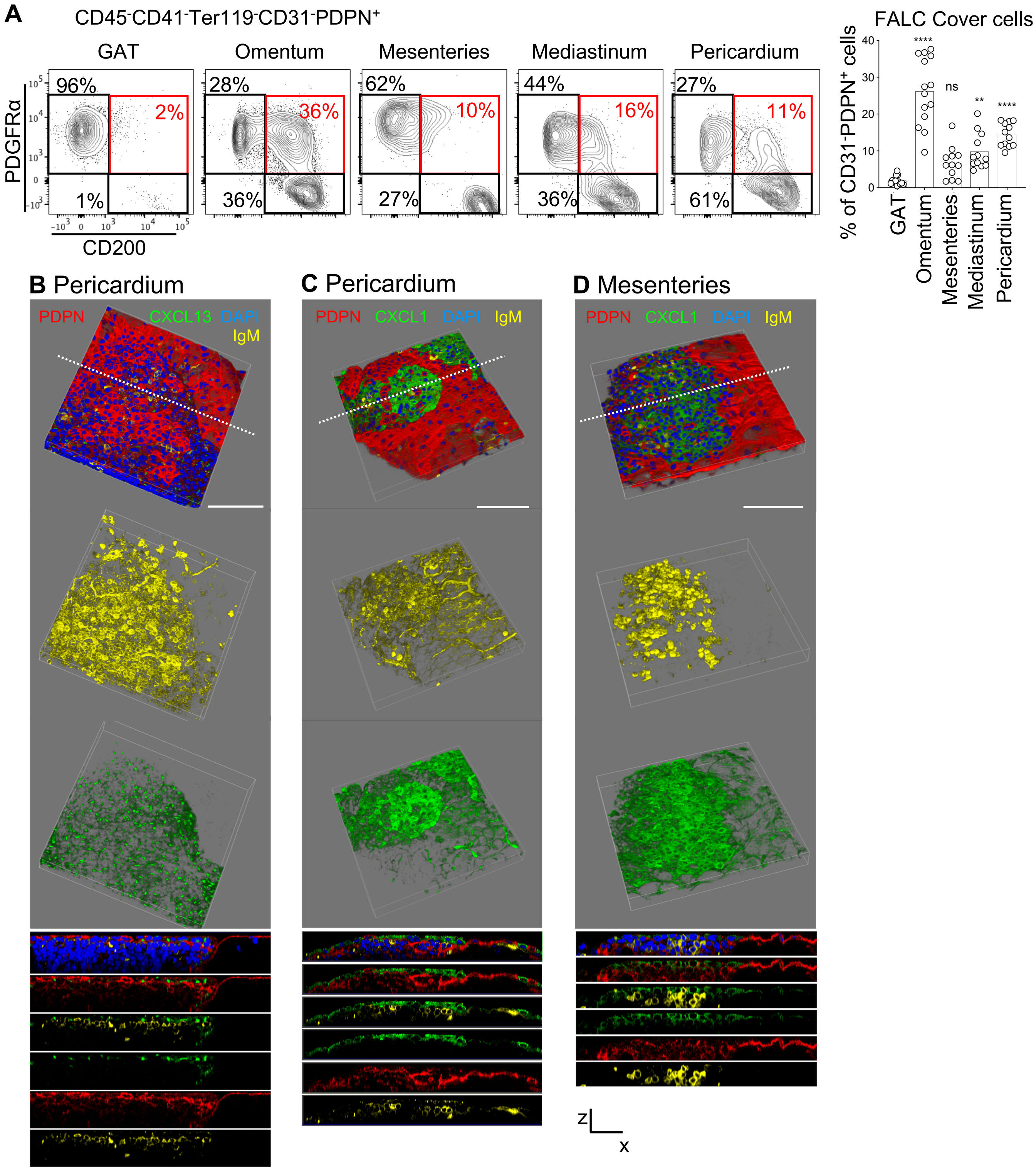

Figure S3. FALC cover cells in other FALC rich visceral adipose tissues related to Figure 1 and 2. A, Representative gating strategy used to determine the percentage of PDPN^{+}PDFGRa^{int}CD200^{int} *Cxcl13*$^{+}$ FALC cover cells (red gate) present in the CD45^{-}CD41^{-}Ter119^{-}CD31^{-}PDPN^{+} non-endothelial stromal cell fraction of the gonadal adipose tissue (GAT), omentum, mesenteries, mediastinum and pericardium. Data pooled from four independent experiments with n=10 mice per group. ANOVA with Sidak's multiple comparisons test were applied after assessing normality using D'Agostino & Pearson Normality test, ns= non-significant, ** P=<0.01, **** P=<0.0001. **B-D,** Representative confocal imaging and 3D reconstruction of a FALC from the pericardium (**B, C**) or the mesenteries (**D**) showing a view of the surface of the cluster and a z section (along the dotted line) of the cluster with PDPN (red), CXCL13 (**B**, green) or CXCL1 (**C**, **D**, green), DAPI (blue) and IgM (red). All staining representative of n≥8 clusters from n≥4 mice in at least 2 independent experiments. Scale bar 100 µm.

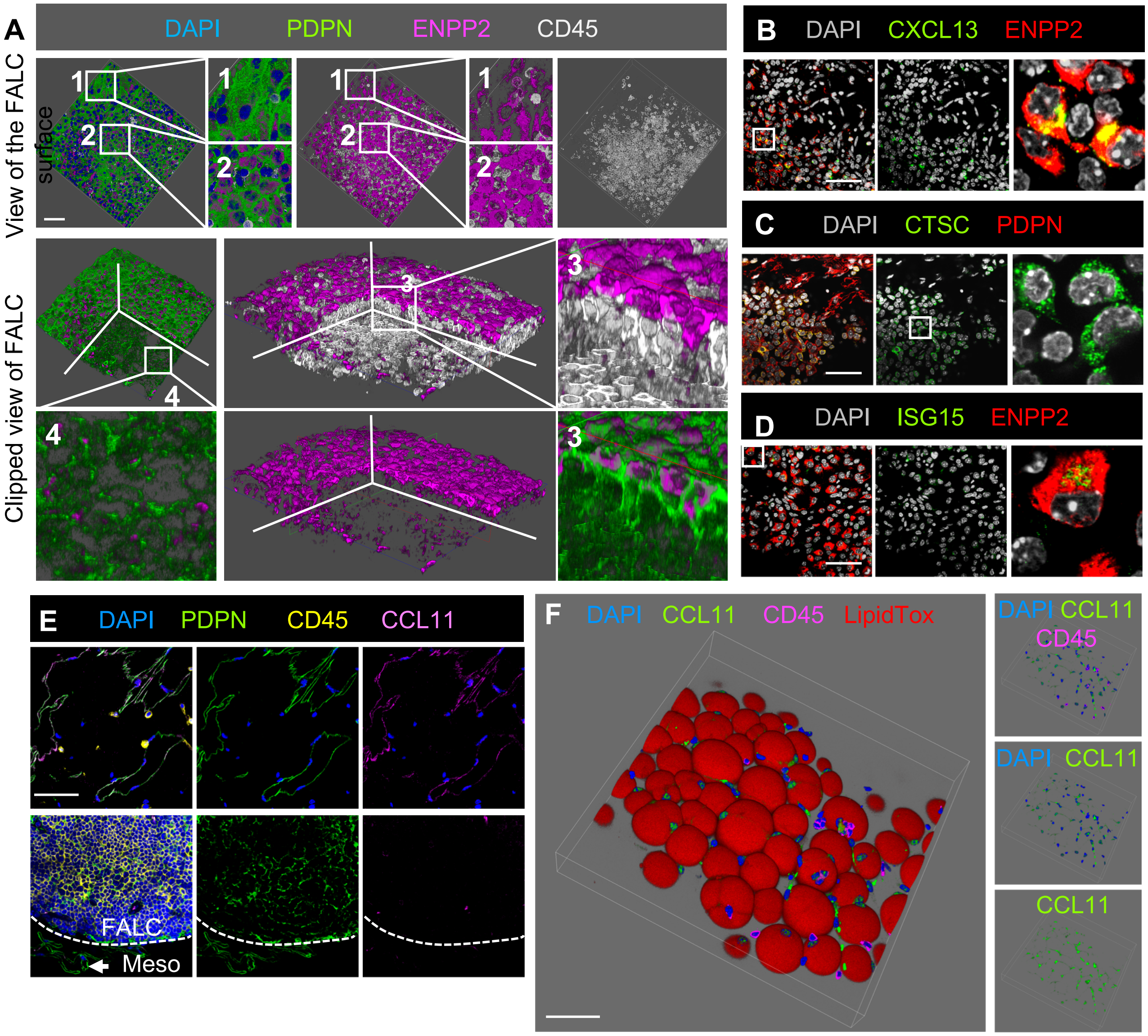

Figure S4. Characterization of FALC stromal cells related to Figure 2. A, 3D reconstruction of omFALC obtained by confocal imaging of wholemount immunofluorescence staining of the omentum showing the surface of the cluster (first row) and two clipped views of the inside of the cluster (second row). Four enlargements are shown: 1 for mesothelial cells, 2 and 3 for FALC cover cells and 4 for FALC FRCs. **B-D,** Representative confocal images of wholemount immuno-fluorescence staining of omentum showing the surface of omFALCs and the expression of CXCL13 (**B**, green), Cathepsin-C (**C**, green) and ISG15 (**D**, green) with DAPI (white), ENPP2 (**A,D** red) and PDPN (**C**, red) by FALC cover cells. Scale Bar 50μM. Staining representative of n≥8 clusters from n≥4 mice in 2 independent experiments. **E,** Representative confocal image of naive whole mount immunofluorescence staining of omentum, DAPI (blue), PDPN (green), CD45 (yellow), CCL11 (magenta). Upper panel showing omental adipose in the absence of FALC, lower panel $CD45^+$ FALC within omental adipose. FALC boundary delineated by dotted line in lower panel. Scale bar 50μM. **F**, Representative confocal imaging and 3D reconstruction of adipocyte in omentum with neutral lipid stained with LipidTox (red), CCL11 (green), DAPI (blue) and CD45 (magenta). Scale bar 100 μm. Staining representative of n=8 clusters or field of view from n=4 mice in 2 independent experiments.

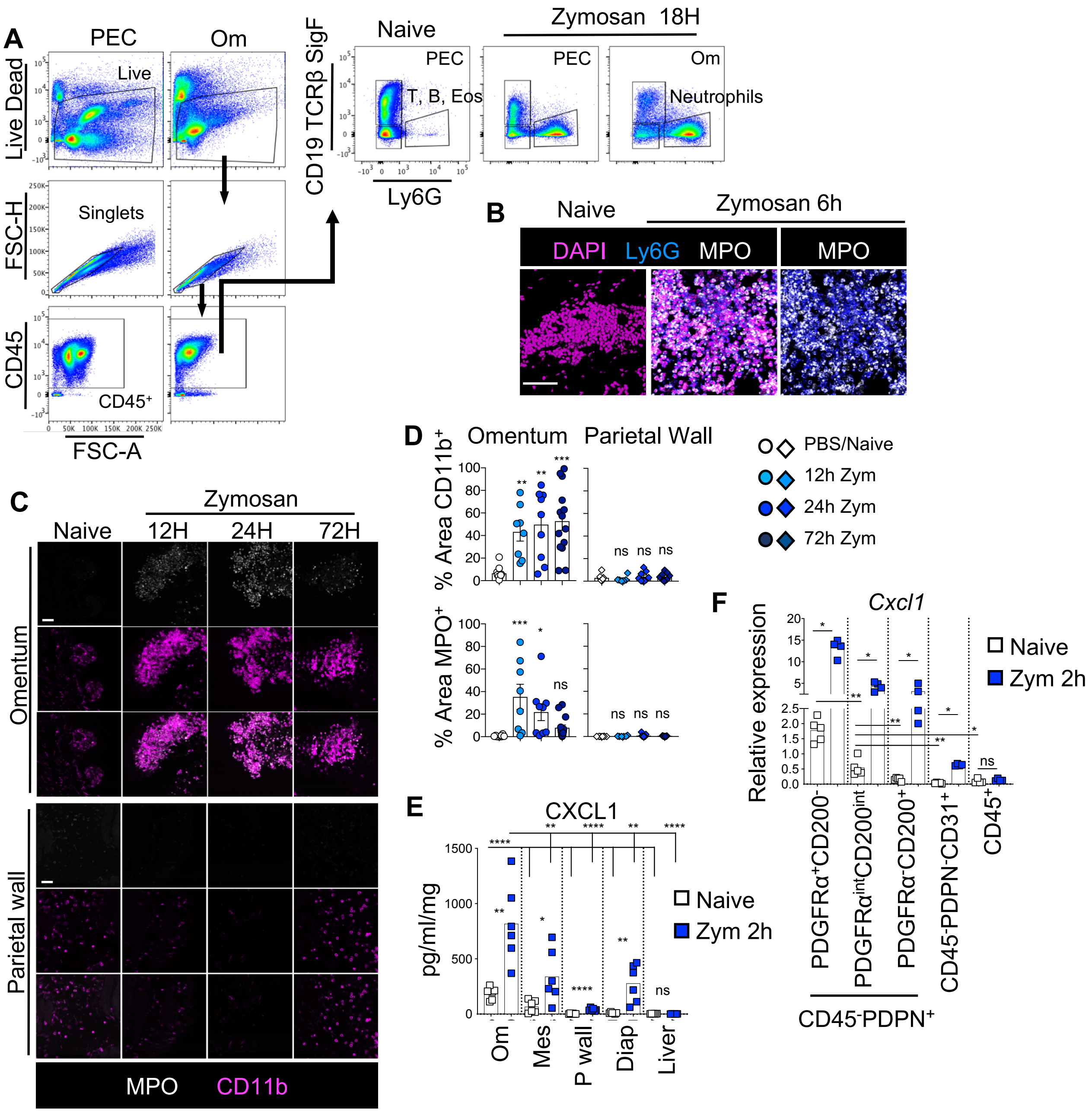

Figure S5. Analysis of neutrophil recruitment in the peritoneal cavity following Zymosan-A injection related to Figure 3. A, Flow-cytometric gating strategy of murine PEC and omentum digests. PEC and omentum were analysed by dead cell exclusion, determination of single cell populations (including B cells) using scatter profiles and based on CD45 positivity. $CD19^{+}$ B cells, $TCR\beta^{+}$ T cells and Siglec F^{+} eosinophils were excluded, $Ly6G^{+}$ neutrophils were gated. **B**, Representative confocal images of wholemount immuno-fluorescence staining of omentum from naïve mice and 6 hours post-i.p. injection of Zymosan-A, DAPI (magenta), MPO (White) and Ly6G (Blue). **C-D**, Representative confocal images of wholemount immuno-fluorescence staining of the omentum and the peritoneal surface of the parietal wall from naïve mice and at the indicated time points following i.p. injection of Zymosan with MPO (white) and CD11b (magenta) (**C**) and quantification of the percentage $CD11b^{+}$ or MPO^{+} area (**D**). **E**, Amounts of CXCL1 secreted into the supernatant of 2h omentum, mesenteric, parietal wall, diaphragm or liver explant culture per mg of tissue and per ml from naïve mice (white squares) and 2h after i.p. injection of Zymosan-A (blue squares). Data pooled from two independent experiments with n=6 mice per group. **F**, Relative amounts of *Cxcl1* expressed by the following stromal cell populations ($CD45^{-}CD41^{-}Ter119^{-}$) $PDPN^{+}CD31^{-}PDGFRa^{+}CD200^{-}$, $PDPN^{+}CD31^{-}PDGFRa^{int}CD200^{int}$, $PDPN^{+}CD31^{-}PDGFRa^{-}CD200^{+}$, $CD31^{+}$, and $CD45^{+}$ hematopoietic cells isolated from naïve mice and 2h post-Zymosan A i.p. injection. Data pooled from 2 independent experiments with n=4 biological replicates per group. ANOVA with Sidak's multiple comparisons test (**D**, **E**) or Mann Whitney test (**F**) were applied after assessing normality using Shapiro-Wilk Normality test, ns= non-significant, **** P=<0.0001. All staining representative of n≥8 clusters or areas from n≥4 mice in at least 2 independent experiments. Scale bar 50 μm.

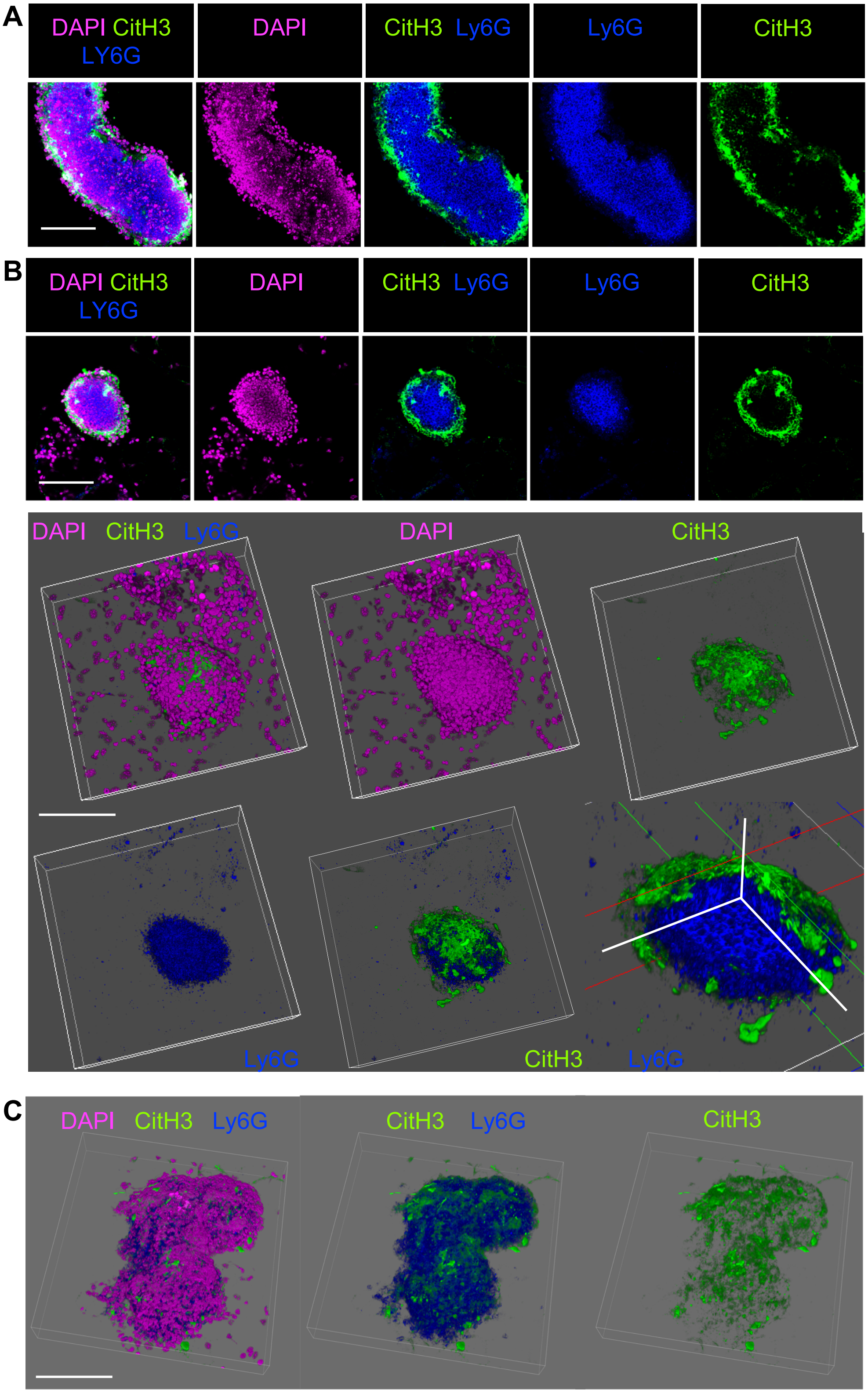

Figure S6. CitH3^{+} DNA covers neutrophil aggregates forming on omental and mesenteric FALCs during peritonitis related to Figure 4. A, Confocal image showing inside the cluster presented in Fig. 4C obtained by wholemount immuno-fluorescence staining of omentum from mice 6 hours post-Zymosan injection showing DAPI (magenta), CitH3 (Green) and Ly6G (Blue). **B**, Confocal image showing inside another representative cluster 6 hours post-Zymosan injection (first panel) and 3D reconstruction of whole omFALC showing the surface of the cluster (first row) and a clipped view of the inside of the cluster (second row). **C**, Representative 3D reconstruction obtained by wholemount immuno-fluorescence staining of mesenteries from mice 6 hours post-Zymosan injection as in **A**. All staining representative of n≥8 clusters or areas from n≥4 mice in at least 2 independent experiments. Scale bar 100µM.

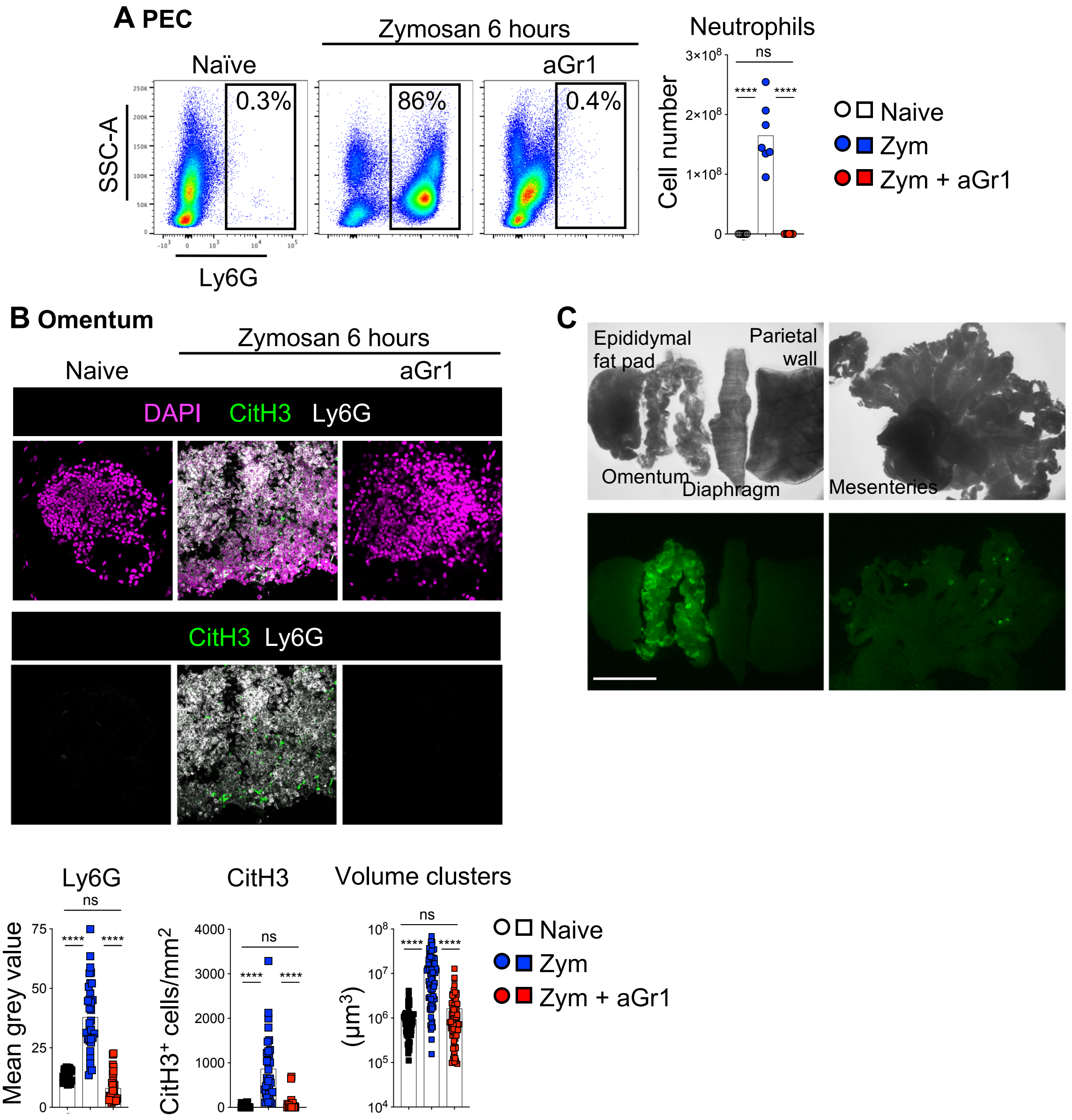

Figure S7. Neutrophil depletion blocks the formation of aggregates recovered with CitH3^{+} DNA and FALCs concentrates Fluo-Zym particles in the omentum and mesenteries related to Figure 4 and 5. **A**, Mice received anti-Gr1 blocking antibodies to deplete neutrophils 24h prior to Zymosan i.p. injection. Flow-cytometric analysis showing frequency of neutrophils in PEC and quantification of the number of neutrophil found in PEC of naïve mice (white circle) , mice 6h post Zymosan-A injection (blue circle) and mice which received anti-Gr1 antibody prior to Zymosan injection (red circle). Data pooled from two independent experiments with n=6 mice per group. **B**, Representative confocal images of wholemount immuno-fluorescence staining of omFALCs from mice as described in **A** with DAPI (magenta), CitH3 (green) and Ly6G (grey); quantification of the mean grey value of Ly6G and the number of CitH3^{+} nuclei in omFALC sections; and quantification of the volume of omFALCs as assessed by measuring the maximal length, width and depth of clusters visualized with DAPI. Data pooled from two independent experiments with $n \geq 25$ (mean grey value Ly6G and CitH3^{+} quantification) or $n \geq 50$ (cluster size quantification) imaged clusters from n=7 mice per group. **C,** Mice were injected i.p. with Fluo-Zym and the epididymal fat pad, omentum, parietal wall, diaphragm and mesenteries were analyzed 6 hours post injection. Representative stereo-microscope imaging of the tissues showing bright field (top) and Fluo-Zym (bottom). Scale bar 0.5 cm. Data pooled from two independent experiments with n=6 mice per group.

	Pathway	$-Log_{10}(P)$
Matn2⁺ fibroblasts	Elastic fibre formation	1.43891036
	Extracellular matrix organization	1.44523816
	Diseases associated with O-glycosylation of proteins	1.45319981
	Post-translational protein phosphorylation	1.53030527
	Molecules associated with elastic fibres	1.64886348
	IRE1alpha activates chaperones	1.65319331
	O-glycosylation of TSR domain-containing proteins	2.37862719
	Extrinsic Pathway of Fibrin Clot Formation	2.38400989
Ccl11⁺ fibroblasts	Signaling by Interleukins	3.23957752
	Laminin interactions	3.24336389
	Elastic fibre formation	4.17783192
	Collagen formation	4.75945075
	Assembly of collagen fibrils and other multimeric structures	4.7878124
	Integrin cell surface interactions	5.5214335
	Non-integrin membrane-ECM interactions	6.01233374
	Collagen biosynthesis and modifying enzymes	6.03151705
	Regulation of Insulin-like Growth Factor (IGF) transport and uptake by Insulin-like Growth Factor Binding Proteins (IGFBPs)	6.10568394
	Collagen chain trimerization	6.24795155
	Collagen degradation	6.43651891
	Degradation of the extracellular matrix	8.326058
	Axon guidance	8.4034029
	Interleukin-4 and Interleukin-13 signaling	8.82681373
	Extracellular matrix organization	10.4546929
Ifit⁺ mesothelium	Cytokine Signaling in Immune system	1.82320711
	Antiviral mechanism by IFN-stimulated genes	2.31609774
	ISG15 antiviral mechanism	2.57665941
	Regulation of IFNA signaling	2.90374714
	Interferon alpha/beta signaling	7.04000516
	Interferon Signaling	8.65560773
Cxcl13⁺ mesothelium	Interleukin-1 family signaling	2.72327862
	Interferon gamma signaling	2.73810127
	Interleukin-1 signaling	2.79221515
	NIK-->noncanonical NF-kB signaling	2.86431568
	TNFR2 non-canonical NF-kB pathway	2.88167673
	Innate Immune System	3.14327111
	Activation of NF-kappaB in B cells	3.2313619
	Immune System	3.3705904
	Attenuation phase	3.37882372
	Interferon alpha/beta signaling	3.74472749
	Chemokine receptors bind chemokines	3.79317412
	Interferon Signaling	3.95467702
	Signaling by Interleukins	4.84163751
	Cytokine Signaling in Immune system	4.91009489
	Interleukin-10 signaling	6.47366072
Mesothelium	Extracellular matrix organization	2.68782751
	Proton-coupled monocarboxylate transport	2.76531065
	Hemostasis	2.87661033
	Keratan sulfate biosynthesis	3.08196966
	Cell junction organization	3.26042766
	Transport of connexons to the plasma membrane	4.01954211
	Microtubule-dependent trafficking of connexons from Golgi to the plasma membrane	4.1090204
	Cell-Cell communication	4.16941133
	Regulation of Insulin-like Growth Factor (IGF) transport and uptake by Insulin-like Growth Factor Binding Proteins (IGFBPs)	4.34582346
	Formation of tubulin folding intermediates by CCT/TriC	4.51999306
	Gap junction assembly	4.74472749
	Gap junction trafficking and regulation	4.79048499
	Post-chaperonin tubulin folding pathway	4.95860731
	Gap junction trafficking	5.01818139
	Smooth Muscle Contraction	7.81815641

Table S1. Pathway analysis of omentum stromal cell subsets related to Figure 1. Bioreactome analysis of DEGs for each cluster.

	Acute appendicitis	Biliary colic
**Number of participants**	13	10
**Female:Male**	7:6	8:2
**Median age in years (range)**	28 (16 – 58)	48 (22 to 67)
**Body mass index (mean)**	29.1	32.3
**White blood cell count x 10^9/L***	14.78 (5.2 - 19.7)	6.81 (3.90 - 9.80)
**Neutrophil count x 10^9/L***	11.95 (3.16 - 16.81)	4.05 (2.16 - 8.08)
**Lymphocyte count x 10^9/L***	1.63 (0.38 - 2.90)	1.99 (1.13 - 2.74)
**Monocyte count x 10^9/L***	1.10 (0.14 - 2.06)	0.54 (0.35 - 0.75)
**Eosinophil count x 10^9/L***	0.07 (0.01 - 0.06)	0.24 (0.05 - 62.00)
**Basophil count x 10^9/L***	0.02 (0.01 - 0.06)	0.03 (0.01 - 0.05)
**Serum C-reactive protein mg/L**	63 (6 to 215)	4 (< 1 to 5)

*data are mean (range)

Table S2. Summary table of patient demographics related to Figure 7. Sex, Age, body mass index (BMI) white blood cell (WBC) count and C-reactive protein (CRP) characteristics of the Biliary colic patients and acute appendicitis patients recruited for the study.
