# Supplementary material for: Stromal Cells Covering Omental Fat-Associated Lymphoid Clusters Trigger Formation of Neutrophil Aggregates to Capture Peritoneal Contaminants
Source: Immunity. 2020 Apr 14;52(4):700–715.e6. doi: 10.1016/j.immuni.2020.03.011 (PMC7156918; doi:10.1016/j.immuni.2020.03.011)
Supplement: Document S2. Article plus Supplemental Information [file mmc5.pdf]

Immunity

Article

Stromal Cells Covering Omental Fat-Associated Lymphoid Clusters Trigger Formation of Neutrophil Aggregates to Capture Peritoneal Contaminants

Graphical Abstract

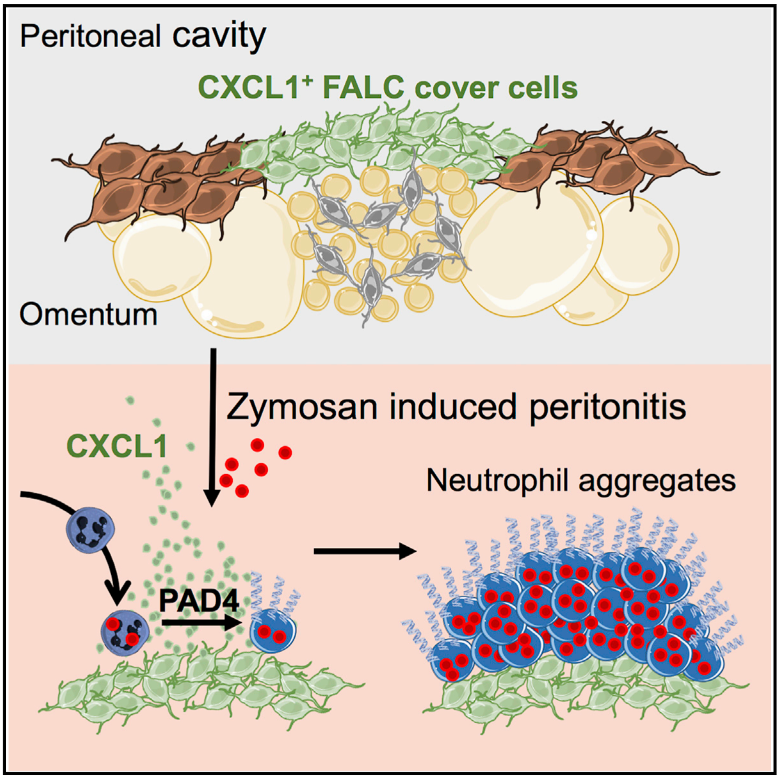

Authors

Lucy Helen Jackson-Jones, Peter Smith, Jordan Raymond Portman, ..., Neil Cowan Henderson, Damian James Mole, Cécile Bénézech

Correspondence

l.jackson-jones@lancaster.ac.uk (L.H.J.-J.), cbenezec@ed.ac.uk (C.B.)

In Brief

For decades, the omentum has been known as the policeman of the abdomen. Jackson-Jones et al. reveal the existence of specialized mesothelial cells within fat-associated lymphoid clusters of the omentum that are responsible for the capture of peritoneal contaminants via the formation of neutrophil aggregates, preventing systemic spread.

Highlights

- Specialized *Cxcl13*⁺ mesothelial cells cover the surface of FALCs and secrete CXCL1
- CXCL1 mediates the recruitment of neutrophils to omental FALCs during peritonitis
- PAD4-dependent neutrophil aggregates mediate the capture of zymosan by the omentum
- Neutrophils form NETs on the omentum of patients with appendicitis

Jackson-Jones et al., 2020, Immunity *52*, 700–715
April 14, 2020 © 2020 The Author(s). Published by Elsevier Inc.
https://doi.org/10.1016/j.immuni.2020.03.011

CellPress

Immunity
Article

Stromal Cells Covering Omental Fat-Associated Lymphoid Clusters Trigger Formation of Neutrophil Aggregates to Capture Peritoneal Contaminants

Lucy Helen Jackson-Jones,[1,2,*] Peter Smith,[1] Jordan Raymond Portman,[3] Marlène Sophie Magalhaes,[1] Katie Jude Mylonas,[1,3] Matthieu Marie Vermeren,[4] Mark Nixon,[1] Beth Emily Pollot Henderson,[3] Ross Dobie,[3] Sonja Vermeren,[3] Laura Denby,[1] Neil Cowan Henderson,[3,5] Damian James Mole,[3] and Cécile Bénézech[1,6,*]
[1]Centre for Cardiovascular Science, University of Edinburgh, Edinburgh EH16 4TJ, UK
[2]Division of Biomedical and Life Sciences, Lancaster University, Lancaster LA1 4YQ, UK
[3]Centre for Inflammation Research, University of Edinburgh, Edinburgh EH16 4TJ, UK
[4]Centre for Regenerative Medicine, University of Edinburgh, Edinburgh EH16 4TJ, UK
[5]MRC Human Genetics Unit, Institute of Genetics and Molecular Medicine, University of Edinburgh, Edinburgh EH4 2XU, UK
[6]Lead Contact
*Correspondence: l.jackson-jones@lancaster.ac.uk (L.H.J.-J.), cbenezec@ed.ac.uk (C.B.)
https://doi.org/10.1016/j.immuni.2020.03.011

SUMMARY

The omentum is a visceral adipose tissue rich in fat-associated lymphoid clusters (FALCs) that collects peritoneal contaminants and provides a first layer of immunological defense within the abdomen. Here, we investigated the mechanisms that mediate the capture of peritoneal contaminants during peritonitis. Single-cell RNA sequencing and spatial analysis of omental stromal cells revealed that the surface of FALCs were covered by CXCL1$^+$ mesothelial cells, which we termed FALC cover cells. Blockade of CXCL1 inhibited the recruitment and aggregation of neutrophils at FALCs during zymosan-induced peritonitis. Inhibition of protein arginine deiminase 4, an enzyme important for the release of neutrophil extracellular traps, abolished neutrophil aggregation and the capture of peritoneal contaminants by omental FALCs. Analysis of omental samples from patients with acute appendicitis confirmed neutrophil recruitment and bacterial capture at FALCs. Thus, specialized omental mesothelial cells coordinate the recruitment and aggregation of neutrophils to capture peritoneal contaminants.

INTRODUCTION

The omentum, a visceral fat depot contained within a fold of peritoneum, has the capacity to rapidly absorb particles and pathogens present in the peritoneal cavity (Meza-Perez and Randall, 2017; Platell et al., 2000). The omentum has important immunological properties derived from the presence of numerous immune cell clusters called fat-associated lymphoid clusters (FALCs; Rangel-Moreno et al., 2009), which are also found in the mesentery (Moro et al., 2010), the mediastinum, and pericardium, in association with the peritoneal, pleural, and pericardial cavities (Bénézech et al., 2015; Jackson-Jones et al., 2016). The continuous flow of fluid from the peritoneal cavity through omental (om)FALCs makes them unique niches for the clearance of peritoneal contaminants and initiation of protective immune responses during peritonitis.

FALCs support multifaceted stromal-immune cell interactions, which are critical for the maintenance and function of innate-like B cells (IBCs) within the serous cavities, as well as facilitating T-cell-dependent B cell immune responses to peritoneal antigens (Ansel et al., 2002; Bénézech et al., 2015; Rangel-Moreno et al., 2009). FALC stromal cells produce the chemokine CXCL13, which maintains peritoneal IBCs (Ansel et al., 2002; Bénézech et al., 2015; Rangel-Moreno et al., 2009). Upon inflammatory signals, serous B cells migrate into FALCs where the provision of interleukin (IL)-5 by type 2 innate lymphocytes (ILC2s) causes rapid B cell proliferation and IgM secretion (Jackson-Jones and Bénézech, 2018; Jackson-Jones et al., 2016). FALC stromal cells produce IL-33 (Jackson-Jones et al., 2016), which induces IL-5 secretion by ILC2s (Moro et al., 2010). Peritonitis induces *de novo* FALC formation that is dependent on the production of tumor necrosis factor (TNF) by monocytes and/or macrophages, and TNF receptor (TNFR) signaling in stromal cells (Bénézech et al., 2015). The initial recruitment of inflammatory monocytes into FALCs requires MYD88 dependent activation of *Ccl19*-expressing FALC stromal cells (Perez-Shibayama et al., 2018). The cross-talk between monocytes and FALC stromal cells supports B cell differentiation (Perez-Shibayama et al., 2018) and FALC expansion (Bénézech et al., 2015).

FALCs are highly vascularized (Cruz-Migoni and Caamaño, 2016; Meza-Perez and Randall, 2017) and act as gateways to the peritoneal cavity during peritonitis. During TNF-induced peritonitis, neutrophils rapidly transit through the high endothelial venules (HEVs) of omFALCs to enter the peritoneal cavity (Buscher et al., 2016). The extravasation of cells into the peritoneal cavity via FALCs may be facilitated by the presence of a loose lining of mesothelial cells (Doherty et al., 1995; Hodel, 1970).

The ability of the omentum to collect peritoneal contaminants is attributed to the flow of fluid from the peritoneal cavity through the omentum, with omFALCs acting as an integrated filtration

This is an open access article under the CC BY license (http://creativecommons.org/licenses/by/4.0/).

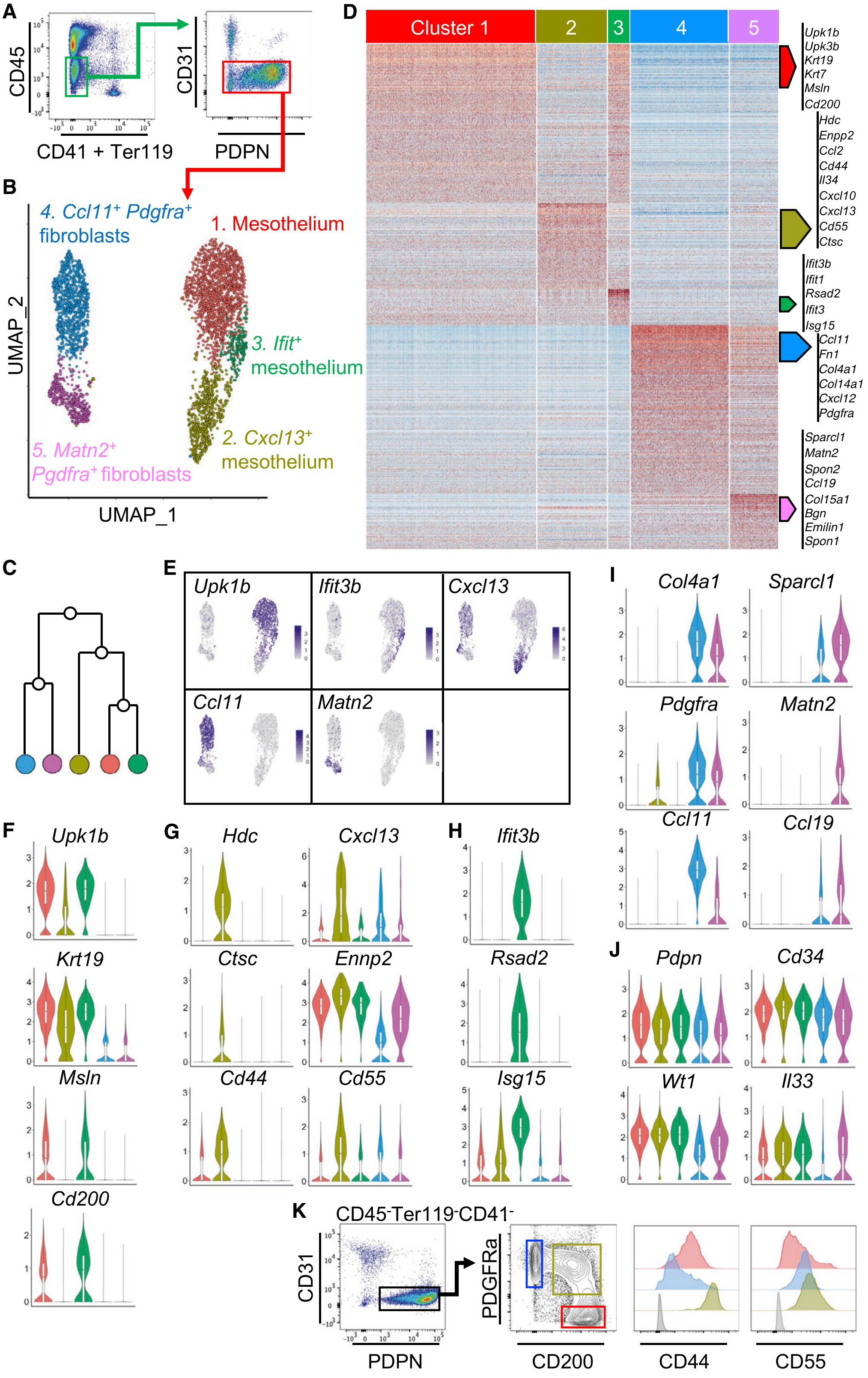

(legend on next page)

system. Here, we investigated the mechanisms enabling the capture of peritoneal contaminants by FALCs by first defining the contribution of stromal-immune cell interactions to the capture and neutralization of contaminants. Single-cell RNA sequencing (scRNA-seq) revealed heterogeneity within the stomal compartment and defined two populations of mesothelial-derived stromal cells. Three-dimensional reconstruction of FALC stromal architecture showed that mesothelial-derived stromal cells covered the surface of omFALCs. These cells produced immune mediators including the neutrophil recruitment chemokine, CXCL1. CXCL1 was critical for the retention and accumulation of neutrophils in omFALCs during Zymosan-induced peritonitis. Neutrophil aggregates at omFALCs were coated with neutrophil extracellular trap (NET)-like DNA structures that concentrated Zymosan particles. *In vivo* chemical inhibition of protein arginine deiminase 4 (PAD4), an enzyme important for NET formation, abolished neutrophil aggregation at omFALCs and resulted in increased dissemination of peritoneal contaminants to the spleen. Similar NET-like DNA structures were detected within the omentum of patients with acute appendicitis. Thus, stromal cells within omFALCs coordinate the neutrophil response to restrict peritoneal contaminants. Manipulating this pathway may provide therapeutic avenues for the treatment of peritonitis.

RESULTS

scRNA-Seq Reveals the Presence of Three Distinct Omental FALC Mesothelial Cell Populations

To characterize the mesothelial and stromal cell populations of the omentum, we performed droplet-based scRNA-seq on isolated mouse omental CD45$^-$CD41$^-$Ter119$^-$CD31$^-$PDPN$^{+/-}$ stromal cells from naive mice (Figure 1A). Unsupervised clustering identified five populations visualized using UMAP (uniform manifold approximation and projection) and a hierarchical cluster tree (Figures 1B and 1C). Cluster 1 was designated as mesothelial cells because differentially expressed genes (DEGs; genes with a 0.25 log-fold change and expressed in at least 25% of the cells in the cluster under comparison; Table S3) were enriched for epithelial (*Upk1b*, *Upk3b*, *Krt19*, and *Krt7*) and mesothelial (*Msln* and *Cd200*) lineage marker genes (Figures 1D–1F and S1A). Clusters 2 and 3 (Figures 1B and 1C) shared similarity with mesothelial cells in keeping with the expression of some epithelial lineage marker genes such as *Upk1b*, *Upk3b*, *Krt19*, and *Krt76* (Figures 1F and S1A). Cluster 2 was distinguished by DEGs involved in the recruitment, adhesion, or activation of immune cells such as *Hdc*, *Enpp2*, *Ccl2*, *Cd44, Il34*, *Cxcl10*, *Cxcl13*, *Cd55*, *Ctsc*, *Ccl7*, and *Cxcl1* and was designated *Cxcl13*$^+$ mesothelium (Figures 1D, 1E, 1G, and S1B). A population of CXCL13$^+$ stromal cells is found around the outside of FALCs (Bénézech et al., 2015; Rangel-Moreno et al., 2009). The fact that *Cxcl13*$^+$ mesothelial cells expressed mesothelial markers suggested that *Cxcl13*$^+$ cells were covering the surface of FALCs. Cluster 3 was distinguished by DEGs associated with interferon signaling such as *Ifit3b*, *Ifit3*, *Ifit1*, and *Isg15*, and anti-viral responses such as *Rsad2*, and was designated as *Ifit*$^+$ mesothelium (Figures 1D, 1E, 1H, and S1C). Pathway analysis confirmed association of this cluster with interferon signaling and anti-viral mechanism terms (Table S1). Pseudotime analysis of the mesothelial cell cluster (cluster 1) to the *Cxcl13*$^+$ mesothelial cluster (cluster 2) showed the gradual up and downregulation of groups of genes along the mesothelial to *Cxcl13*$^+$ mesothelial trajectory (Figures S2A and S2C). Pseudotime analysis also revealed groups of genes whose expression were gradually up and downregulated along the mesothelial (cluster 1) to *Ifit*$^+$ mesothelial (cluster 3) trajectory (Figures S2B and S2D). This suggests that cells from the *Cxcl13*$^+$ and *Ifit*$^+$ mesothelial cell clusters derive from mesothelial cells and acquire specific immune functions.

scRNA-Seq Reveals the Presence of Two Distinct FALC Fibroblast Populations within Omental Stroma

Clusters 4 and 5 displayed DEGs enriched for genes associated with fibroblasts such as *Fn1*, *Col4a1*, *Col14a1*, and *Pdgfra* (Figures 1D, 1I, and S1D). Cluster 4 was distinguished by the expression of *Ccl11* (also called Eotaxin) and assigned the name *Ccl11*$^+$*Pdgfra*$^+$ fibroblasts (Figures 1B, 1E, and 1I). Cluster 5 was characterized by the expression of *Matn2.* FALC B cells of the omentum and mesenteries are embedded in a dense network of PDGFRα^+ fibroblast reticular cells (FRCs) expressing *Ccl19* (Perez-Shibayama et al., 2018). *Matn2*$^+$*Pdgfra*$^+$ fibroblasts expressed *Ccl19* and showed enrichment for genes involved in formation of the extracellular matrix (ECM) characteristic of lymph node (LN) and FALC FRCs such *as Sparcl, Spon2, Col15a1, Bgn, Emilin1*, and *Spon1* (Figures 1D, 1I, and S1E) (Huang et al., 2018; Malhotra et al., 2012; Perez-Shibayama et al., 2018). They represented a distinct subset of FRCs, whose gene expression profile did not fit into any of the LN stromal cell populations recently described by scRNA-seq (Rodda et al., 2018). In particular, *Matn2*$^+$*Pdgfra*$^+$ fibroblasts did not express *Il7*, *Ccl21*, *Bst1*, or *Cxcl9*, but expressed high levels of *Cd34* (Figure 1J), *Inmt*, and *Nr4a1* (Figure S1E). The *Matn2*$^+$*Pdgfra*$^+$ fibroblast subset was distinct from the populations of fibroblasts identified in the inflamed synovium (Croft et al., 2019) and did not express *Fapa or Thy1*. *Ccl11*$^+$*Pdgfra*$^+$ fibroblasts did not express *Fapa* and showed only limited expression of *Thy1*

Figure 1. Identification of Non-endothelial Stromal Cell Populations within the Omentum

(A) CD45$^-$CD41$^-$Ter119$^-$CD31$^-$PDPN$^{+/-}$ non-endothelial omental stromal cells were cell-sorted and underwent scRNA-seq.

(B and C) Unsupervised clustering of non-endothelial omental stromal cells visualized with UMAP, where each dot is a single cell colored by cluster assignment (B) and hierarchical cluster tree (C).

(D) Heatmap of each cell's (column) scaled expression of DEGs (row) expressed by a minimum of 30% of cells per cluster.

(E) Gene expression distinguishing the five clusters projected onto UMAP plots. Color scaled for each gene with highest log-normalized expression level noted.

(F–J) Violin plots of canonical omental stromal cell gene expression by cluster with highest log-normalized expression value labeled for the mesothelium (F and J), *Cxcl13*$^+$ mesothelial cells (G), *Ifit*$^+$ mesothelial cells (H), and *Ccl11*$^+$ *Pdgfra*$^+$ and *Matn2*$^+$ *Pdgfra*$^+$ fibroblasts (I).

(K) Representative gating strategy of non-endothelial omental PDPN$^+$PDFGRa$^+$CD200$^-$ (blue), PDPN$^+$PDFGRa^{int}CD200^{int} (green), and PDPN$^+$PDFGRa$^-$CD200$^+$ (red) stromal cells and level of expression of CD44 and CD55 in these populations. Fluorescence minus one control (FMO) in gray.

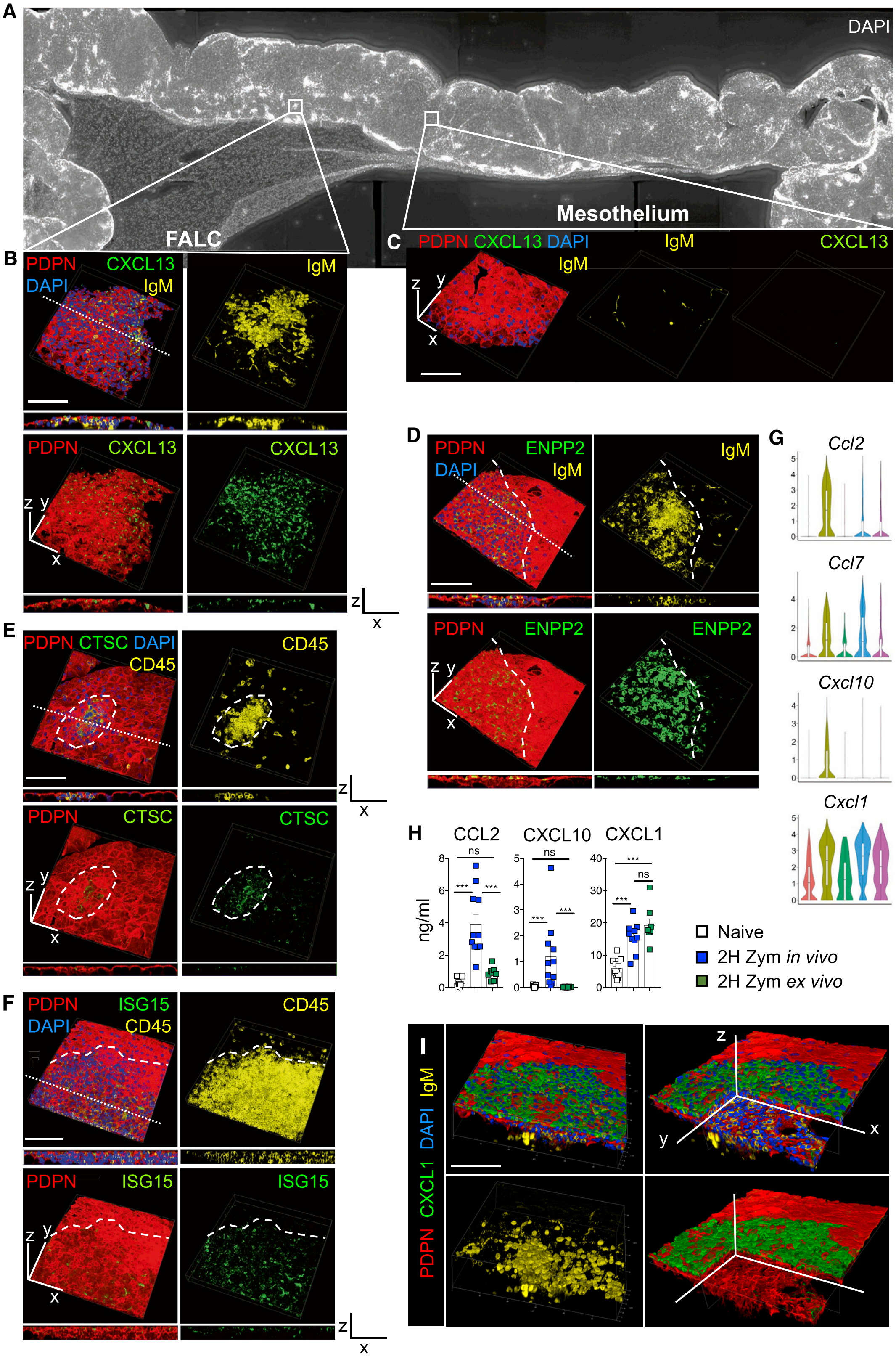

(legend on next page)

(Figure S1D), thus suggesting that the adipose tissue is associated with distinct and specific fibroblast subsets.

All five clusters expressed *Pdpn*, *Wt1*, *Cd34*, *Itgb1* (*Cd29*), and *Ly6a* (*Sca-1*), which have been used collectively to identify cells of mesothelial origin giving rise to adipocytes in visceral fat depots (Figures 1J and S1F; Chau et al., 2014). Regulation of retinol metabolism by *Wt1*-expressing cells is critical to maintain GATA6$^+$ resident macrophages in the peritoneal cavity (Buechler et al., 2019). The two step-limiting enzymes of retinol metabolism were expressed by omental stromal cells with expression of *Aldh1a1* by mesothelial cells and *Aldh1a2* by both mesothelial cells and *Ccl11*$^+$*Pdgfra*$^+$ fibroblasts (Figure S1F). *Il33* was expressed by all three mesothelial clusters (Figure 1J), in agreement with previous reports (Mahlakõiv et al., 2019; Spallanzani et al., 2019) as well as in FALC FRCs, confirming our previous observation that IL-33 is expressed by FALC stromal cells (Jackson-Jones et al., 2016).

Cxcl13$^+$ and *Ifit*$^+$ Mesothelial Cells Are Present in Other Adipose Tissues Rich in FALCs

Distinct clusters of PDPN$^+$PDGFRα^+ fibroblasts and PDPN^{+-}PDGFRα^- mesothelial cells are found in the mesenteric adipose tissue (Koga et al., 2018). As mesenteries contain FALCs, we reasoned that cells corresponding to *Cxcl13*$^+$ and *Ifit*$^+$ mesothelial cells should be identifiable within the mesenteric scRNA-seq dataset (Koga et al., 2018). Projection of the mesenteric PDPN$^+$PDGFRα^- mesothelial cell dataset onto our omental stromal dataset confirmed the presence of *Cxcl13*$^+$ and *Ifit*$^+$ mesothelial cells in the mesenteries (Figures S2E–S2G). Projection of the mesenteric PDPN$^+$PDGFRα^+ fibroblasts onto our omental stromal dataset confirmed the presence of *Matn2*$^+$ fibroblast cells in the mesenteries (Figures S2E, S2F, and S2H).

We compared the gene expression profile of PDPN$^+$PDGFRα^- mesothelium from the gonadal adipose tissue, which does not contain FALCs, with the PDPN$^+$PDGFRα^- mesothelium of the omentum using published RNA-seq datasets (Buechler et al., 2019). A number of genes were overexpressed in the omental mesothelium, including DEGs characterizing the *Cxcl13*$^+$ mesothelial cluster such as *Krt76*, *Cd55*, *Cd44*, *Ccl2*, *Hdc, Enpp2*, and *Cxcl1* (Figure S1H; Table S4). In our omental dataset, *Cxcl13*$^+$ mesothelial cells were distinguished from mesothelial cells by 195 DEGs (Table S5). Of these, 68 were also overexpressed in the omental mesothelium compared to the gonadal fat pad mesothelium of the Buechler et al. dataset (Figure S1G), confirming that the *Cxcl13*$^+$ mesothelial cluster was associated with the presence of FALCs in the adipose tissue.

Flow-cytometric analysis of FALC-rich tissues confirmed the presence of PDGFRα^-PDPN$^+$CD200$^+$CD55$^{-/low}$CD44^{low} mesothelial cells and PDGFRα^+PDPN$^+$CD200$^-$CD55^{int}CD44$^-$ fibroblasts in the omentum and allowed the identification of a population of cells transitioning from PDGFRα^-CD200^{high} to PDGFRα^+CD200$^-$ expressing high levels of CD55 and CD44 in keeping with the gene expression profile of *Cxcl13*$^+$ mesothelial cells (Figure 1K). Detection of CD200 on the surface of *Cxcl13*$^+$ mesothelium suggested maintenance of protein expression following *Cd200* transcript downregulation (Figure 1F) during differentiation from mesothelial cells. In mouse, the omentum is the tissue with the highest abundance of FALCs, followed by pericardium, mediastinum, and mesenteries (Bénézech et al., 2015). In keeping with the relative abundance of FALCs in these tissues, the omentum was the tissue with the highest proportion of *Cxcl13*$^+$ mesothelial cells identified as PDPN$^+$PDGFRα^{int} CD200^{int} cells, followed by the pericardium, mediastinum, and mesenteries. The gonadal adipose tissue, which does not contain FALCs, showed minimal PDPN$^+$PDGFRα^{int}CD200^{int} cells (Figure S3A).

FALCs Are Covered by a Monolayer of Stromal Cells Expressing Markers of *Cxcl13*$^+$ and *Ifit*$^+$ Mesothelial Cells

To elucidate the spatial organization of FALCs, we performed wholemount immunofluorescence staining using marker genes identified from our scRNA-seq analysis. The B cell positioning chemokine CXCL13 was found within PDPN$^+$ mesothelial cells covering the surface of omFALCs (Figures 2A and 2B; FALC), as well as pericardial FALCs (Figure S3B). The morphology of these cells differed from the typical cobblestone appearance of PDPN$^+$ mesothelial cells, which surrounded the FALC and did not express CXCL13 (Figures 2A and 2C; mesothelium). The lysophospholipase ENPP2 (Ectonucleotide Pyrophosphatase/Phosphodiesterase 2), expression of which was enriched in the *Cxcl13*$^+$ FALC stromal cell population, was also present at high levels on the surface of FALCs and was expressed by CXCL13$^+$ cells (Figures 2D, S4A, and S4B). The lysosomal cysteine protease Cathepsin-C (*Ctsc*; also known as di-peptidyl peptidase I) was present within PDPN$^+$ stromal cells covering omFALCs, but not within the mesothelial surface outwith the FALC (Figures 2E and S4C). Our results thus confirm the

Figure 2. FALCs Are Covered by a Differentiated Monolayer of Mesothelial Cells

(A) 3D reconstruction of a large portion of the omentum obtained by imaging of wholemount staining of the omentum showing omFALCs with DAPI (white).

(B and C) Confocal imaging and 3D reconstruction of an omFALC showing a view of the surface of the cluster and a z section (along the dotted line) of the cluster (B) and of the surface of the omentum (C) with PDPN (red), CXCL13 (green), DAPI (blue), and IgM (yellow).

(D–F) Representative confocal imaging and 3D reconstruction of an omFALC showing a view of the surface of the cluster and a z section (dotted line) of the cluster with PDPN (red); ENPP2 (D, green), Cathepsin-C (E, CTSC, green), or ISG15 (F, green); DAPI (blue) and IgM (D, yellow); or CD45 (E and F, yellow). The surface of the cluster and the mesothelium are delimited by a hyphenated line.

(G) Violin plots of gene expression of inflammatory chemokines by cluster with highest log-normalized expression value labeled.

(H) Amounts of CCL2, CXCL10, and CXCL1 secreted into the supernatant of 2-h omentum explant culture per omentum and per ml after exposure to Zymosan-A for 2 h either *in vivo* (i.p. injection) or *ex vivo*. Data pooled from two independent experiments with n = 10 mice per group.

(I) Confocal imaging and 3D reconstruction of omFALC showing the surface of omFALCs (first column) and a clipped view inside the cluster (second column). Scale bars, 100 μm.

All staining representative of n ≥ 8 clusters from n ≥ 4 mice in at least two independent experiments. Error bars show SEM. Kruskal Wallis test with Dunn's multiple comparisons test or ANOVA with Sidak's multiple comparisons test was applied after assessing normality using D'Agostino and Pearson normality test, ns = non-significant, ***p = < 0.001.

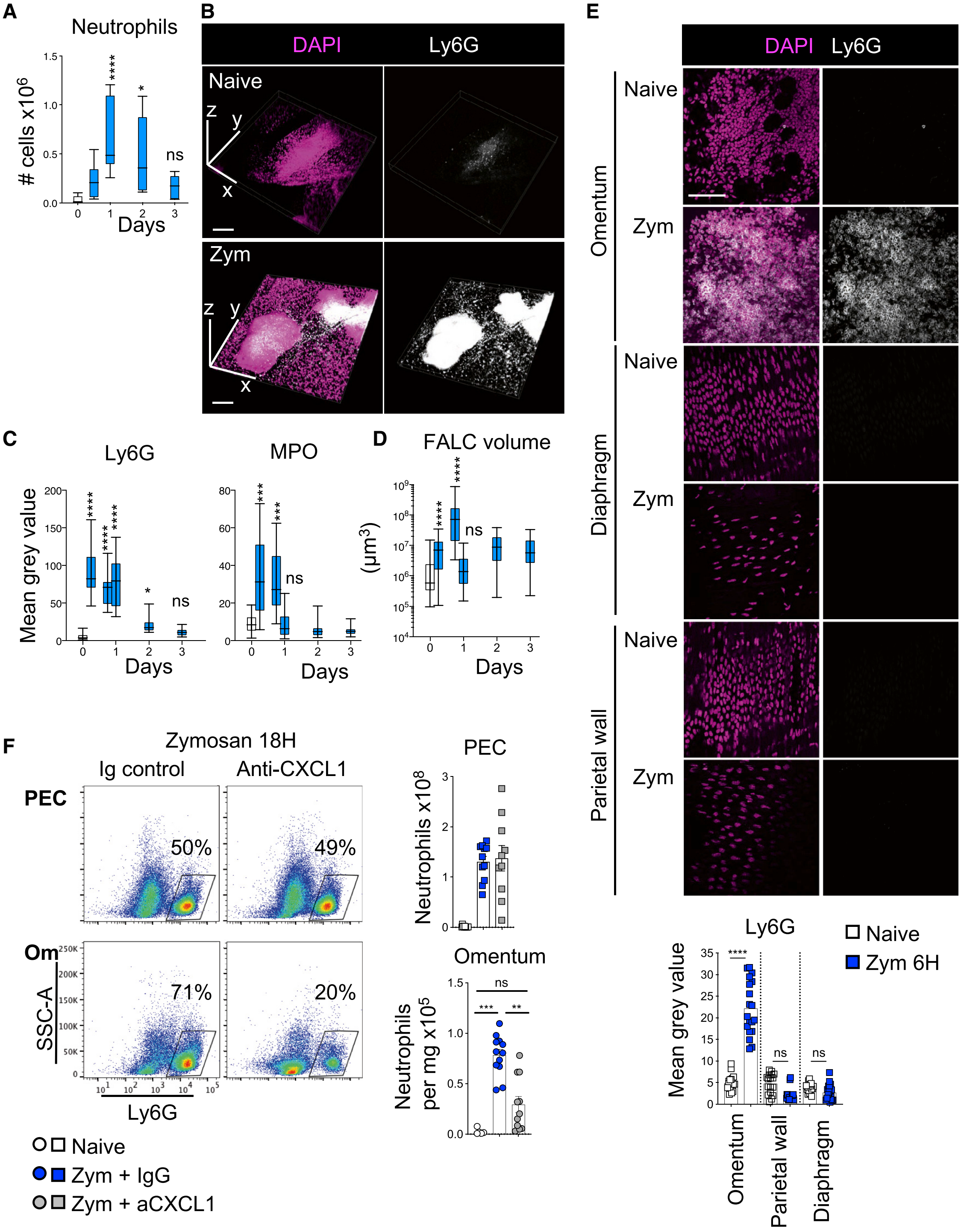

Figure 3. CXCL1 Is Required for the Recruitment of Neutrophils into omFALCs

(A) Number of neutrophils in digested omentum as assessed by flow-cytometric analysis (Figure S5A) of naive (white bar) and at the indicated time points following i.p. injection of Zymosan-A (blue bars). Data pooled from two independent experiments with n = 5–11 mice per group.

(B) Representative confocal imaging and 3D reconstruction of omFALCs from naive and 6 h post-Zymosan mice, DAPI (magenta) and Ly6G (white). Scale bar, 200 μm.

(C) Quantification of the mean gray value of Ly6G and MPO stained as in Figure S5B of omenta from naive (white bar) and at the indicated time points following i.p. injection of Zymosan (blue bars). Data for cluster quantification pooled from two independent experiments with n $\geq$ 24 imaged clusters from n = 8 mice per group.

(D) Quantification of the volume of omFALCs at the indicated times following i.p. injection of Zymosan-A (blue). Data pooled from two independent experiments with n $\geq$ 32 imaged clusters from n = 6 mice per group.

(legend continued on next page)

existence of a mesothelial-derived population of cells covering the surface of FALCs and expressing ENPP2, CXCL13, and Cathepsin-C, which we named *Cxcl13*$^{+}$ FALC cover cells. ISG15 was found in the cytoplasm of a subset of the PDPN^{+} cells covering omFALCs, confirming the existence of *Ifit*$^{+}$ mesothelial cells. ENPP2 and ISG15 were co-expressed by PDPN^{+} cover cells (Figures 2F and S4D). We named these cells *Ifit*$^{+}$ FALC cover cells. PDPN^{+} FALC FRCs, which formed a reticular network at the core of the cluster, expressed very low levels of ENPP2 as predicted (Figure S4A, enlargement 4). Finally, staining for CCL11 revealed that fibroblasts contained in the adipose (non-FALC) stroma of the omentum expressed high levels of CCL11, while FALC FRCs and mesothelial cells did not (Figures S4E and S4F).

Cxcl13^{+} FALC Cover Cells Express Inflammatory Chemokines

The *Cxcl13*$^{+}$ FALC cover cell cluster was distinguished by the expression of the monocyte chemoattractants *Ccl2* and *Ccl7* and the neutrophil chemoattractants *Cxcl1* and *Cxcl10* (Figure 2G), suggesting a role for these cells in orchestrating the recruitment of inflammatory cells during peritonitis. The expression of *Cxcl1* was also enriched in *Ccl11*$^{+}$*Pdgfra*$^{+}$ fibroblasts and *Matn2*$^{+}$*Pdgfra*$^{+}$ fibroblasts. Analysis of omental explant culture supernatants showed that CXCL1 protein was released at steady state by the omentum and that CXCL1 secretion was potentiated after a 2 h exposure to Zymosan-A, *in vivo* or *ex vivo* (Figure 2H). In contrast, the two other early chemo-attractants, CCL2 and CXCL10, were released by the omentum only after peritoneal inflammation was triggered by Zymosan-A, and this could not be recapitulated *ex vivo* (Figure 2H). This suggests that CXCL1 is constitutively produced by the omentum, while the initial secretion of CCL2 and CXCL10 is dependent on the early recruitment of immune cells upon sensing of an inflammatory signal. In addition, the rapid induction of secretion of CXCL1 (2 h) suggests that this is due to release of pre-formed CXCL1 rather than an increase in transcription. Radiation-resistant stromal-derived CXCL1 is important in the control of bacterial infection during peritonitis (Jin et al., 2017). Protein expression of CXCL1 was particularly high in FALC cover cells within the omentum (Figure 2I), as well as the pericardium and mesenteries (Figures S3C and S3D). While the expression of *Cxcl1* was comparable in the *Cxcl13*$^{+}$ mesothelial cell cluster and the *Matn2*$^{+}$*Pdgfra*$^{+}$ and *Ccl11*$^{+}$*Pdgfra*$^{+}$ fibroblast clusters, CXCL1 protein was much higher in FALC cover cells, suggesting that these cells retain intracellular stores of CXCL1. Given the spatially constrained expression of CXCL1 over the surface of FALCs and that neutrophils use FALC HEVs to enter the peritoneal cavity (Buscher et al., 2016), we next assessed whether CXCL1 was important for the recruitment of neutrophils to omFALCs during peritonitis.

CXCL1 Mediates Active Recruitment of Neutrophils into omFALCs

To characterize the dynamics of neutrophil recruitment to omFALCs during peritonitis, we used the well-established murine model, Zymosan-A-induced peritonitis. Peritonitis led to a transient increase in the number of Ly6G^{+} neutrophils in the omentum, peaking at 24 h (Figures 3A and S5A). Wholemount immunofluorescence staining and confocal analysis revealed that FALCs were the site of extensive recruitment of Ly6G^{+} Myeloperoxidase^{+} (MPO) neutrophils, which formed dense cellular aggregates between 6 and 24 h post-induction of peritonitis (Figures 3B, 3C, and S5B). The volume of omFALCs increased exponentially during the first 18 h post-Zymosan injection (75-fold) before rapidly contracting back to their normal size by 24 h (Figure 3D). This timing coincided with the influx of MPO^{+} neutrophils and their disappearance by 24 h (Figures 3C and 3D). This accretion of neutrophils was specific to omFALCs and did not happen on the rest of the surface of the omentum, the diaphragm, or the parietal wall (Figures 3E, S5C, and S5D). We then tested whether CXCL1 was involved in the accumulation of neutrophils at omFALCs during peritonitis, using an anti-CXCL1 blocking antibody. Eighteen hours post-Zymosan injection, CXCL1 blockade led to a 2.6-fold decrease in the number of neutrophils recovered from the omentum compared to mice treated with isotype control antibodies. There was no difference in the number of neutrophils recovered from the peritoneal cavity, suggesting that the trafficking of neutrophils through omFALC HEVs was not altered (Figure 3F). Thus, the extensive recruitment of neutrophils to FALCs is not simply a consequence of increased trafficking of neutrophils through HEVs, but the result of active retention dependent upon CXCL1.

To confirm that the omentum is a major source of CXCL1 during peritonitis, we quantified secretion of CXCL1 following 2 h *ex vivo* explant culture of omentum, mesenteries, peritoneal wall, diaphragm, and liver from naive mice and at 2 h following injection of Zymosan-A. In contrast to liver, the mesenteries, peritoneal wall, and diaphragm all significantly increased secretion of CXCL1 following exposure to Zymosan-A *in vivo*; however, per mg of tissue, the omentum secreted significantly more CXCL1 than any of these peritoneal tissues (Figure S5E). Having established that FALC cover cells constituted an important store of CXCL1, and that inflammation led to rapid release of pre-formed CXCL1, we next assessed the effect of inflammation on the expression of *Cxcl1* mRNA in various omental cell fractions: PDPN^{+}CD31^{-}PDGFRα^{+}CD200^{-} fibroblasts, PDPN^{+}

(E) Representative confocal images of omentum, the peritoneal surface of the diaphragm, and parietal wall from naive and 6 h post-Zymosan mice, DAPI (magenta) and Ly6G (white) and quantification of the mean gray value of Ly6G for omFALCs, parietal wall, diaphragm from naive (white) and 6 h post-Zymosan mice (blue). Data for cluster quantification pooled from two independent experiments with $n \geq 24$ imaged clusters from n = 6 mice per group. Scale bar, 50 μm.
(F) Representative density plots showing proportion of neutrophils found in the peritoneal exudate cells (PECs) and omentum 18 h post Zymosan i.p injection, in combination with the injection of anti-CXCL1 or isotype control antibodies after 2 h and number of neutrophils found in PECs and per g of omentum in naive and treated mice. Data pooled from two independent experiments with n = 5–11 mice per group.
Error bars show SEM. Box and whiskers showing min to max value. Kruskal Wallis test with Dunn's multiple comparisons test or ANOVA with Sidak's multiple comparisons test was applied after assessing normality using D'Agostino and Pearson normality tests, ns = non-significant, * = $p < 0.05$, ** = $p < 0.01$, ***p = < 0.001, ****p = < 0.0001.

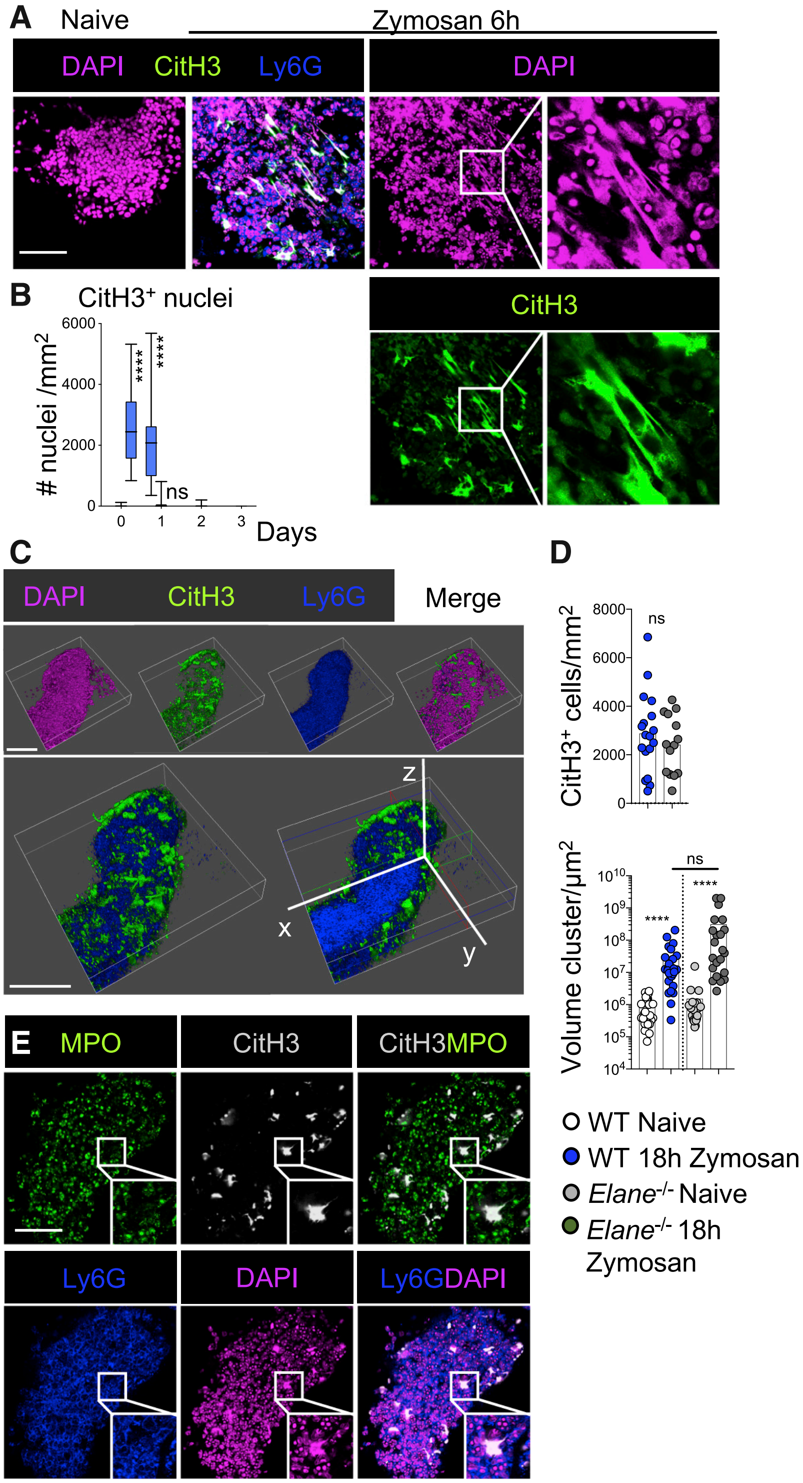

Figure 4. CitH3$^+$ DNA Coats Neutrophil Aggregates on omFALCs during Peritonitis

(A) Representative confocal images of wholemount staining of omentum from naive and 6 h post-Zymosan mice DAPI (magenta), CitH3 (green), and Ly6G (blue). Scale bar, 50 µm.

(B) Quantification of the number of CitH3 positive nuclei per mm at the times indicated post-Zymosan injection. Data for cluster quantification pooled from two independent experiments with n ≥ 24 imaged clusters from n = 8 mice per group. Box and whiskers showing min to max value.

(C) 3D reconstruction of omFALC at 6 h post-Zymosan injection showing the surface of a cluster (first row) and a clipped view inside the cluster (second row). Ly6G (blue), CitH3 (green), DAPI (magenta). Scale bar, 100 µm.

(D) WT or *Elane*$^{-/-}$ mice were left naive (WT white, *Elane*$^{-/-}$ gray) or injected i.p. with Zymosan (WT blue, Elane$^{-/-}$ gray). Omenta were collected 18 h post-injection. The number of CitH3$^+$ cells per mm and volume of each cluster were assessed by wholemount staining and confocal analysis of omenta from naive and treated mice. Data for cluster quantification pooled from two independent experiments with n ≥ 14 (CitH3$^+$ cells) or n ≥ 20 (cluster size) imaged clusters from n = 4 mice per group.

(E) Representative confocal images of omFALCs at 6 h following Zymosan injection showing MPO (green), Ly6G (blue), CitH3 (white), and DAPI (magenta). Staining representative of n ≥ 24 clusters from n ≥ 8 mice in two independent experiments. Scale Bar, 50 µm.

Student's t test, Kruskal Wallis test with Dunn's multiple comparisons test, or Mann Whitney test was applied after assessing normality using D'Agostino and Pearson normality test, ns = non-significant, ****p = < 0.0001.

CD31$^-$PDGFRα^{int}CD200^{int} FALC cover cells, PDPN$^+$CD31$^-$PDGFRα^-CD200$^+$ mesothelial cells, PDPN$^-$CD45$^-$CD31$^+$ endothelial cells, and CD45$^+$ hematopoietic cells. Two hour Zymosan-A exposure significantly increased the transcription of *Cxcl1* by all populations assessed, except the hematopoietic population, where *Cxcl1* expression remained low, confirming the stromal origin of CXCL1 within the omentum. The highest expression of *Cxcl1* was found in fibroblasts and FALC cover cells (Figure S5F).

Neutrophils Form Large Aggregates in omFALCs, Which Are Encapsulated in NET-like Structures

Immunofluorescence imaging analysis of accumulated neutrophils within omFALCs revealed the presence of multiple areas of DAPI staining presenting with a non-nuclear appearance (Figure 4A, right). Neutrophils have the capacity to form NETs, which consist of uncoiled DNA scaffold decorated with proteases and anti-microbial peptides found in neutrophil granules. NETs are formed as a defense mechanism to immobilize invading microorganisms but also in response to sterile triggers (Boeltz et al., 2019; Papayannopoulos, 2018). In some conditions, NETs mediate neutrophil aggregation (Leppkes et al., 2016; Muñoz et al., 2019; Schauer et al., 2014). Chromatin decondensation, which is required for the formation of NETs, can be mediated by the citrullination of histones. Staining for citrullinated histone H3 (CitH3) revealed that the areas of DAPI staining presenting with an extruded DNA pattern were highly stained for CitH3 (Figure 4A). CitH3$^+$ DNA was found in

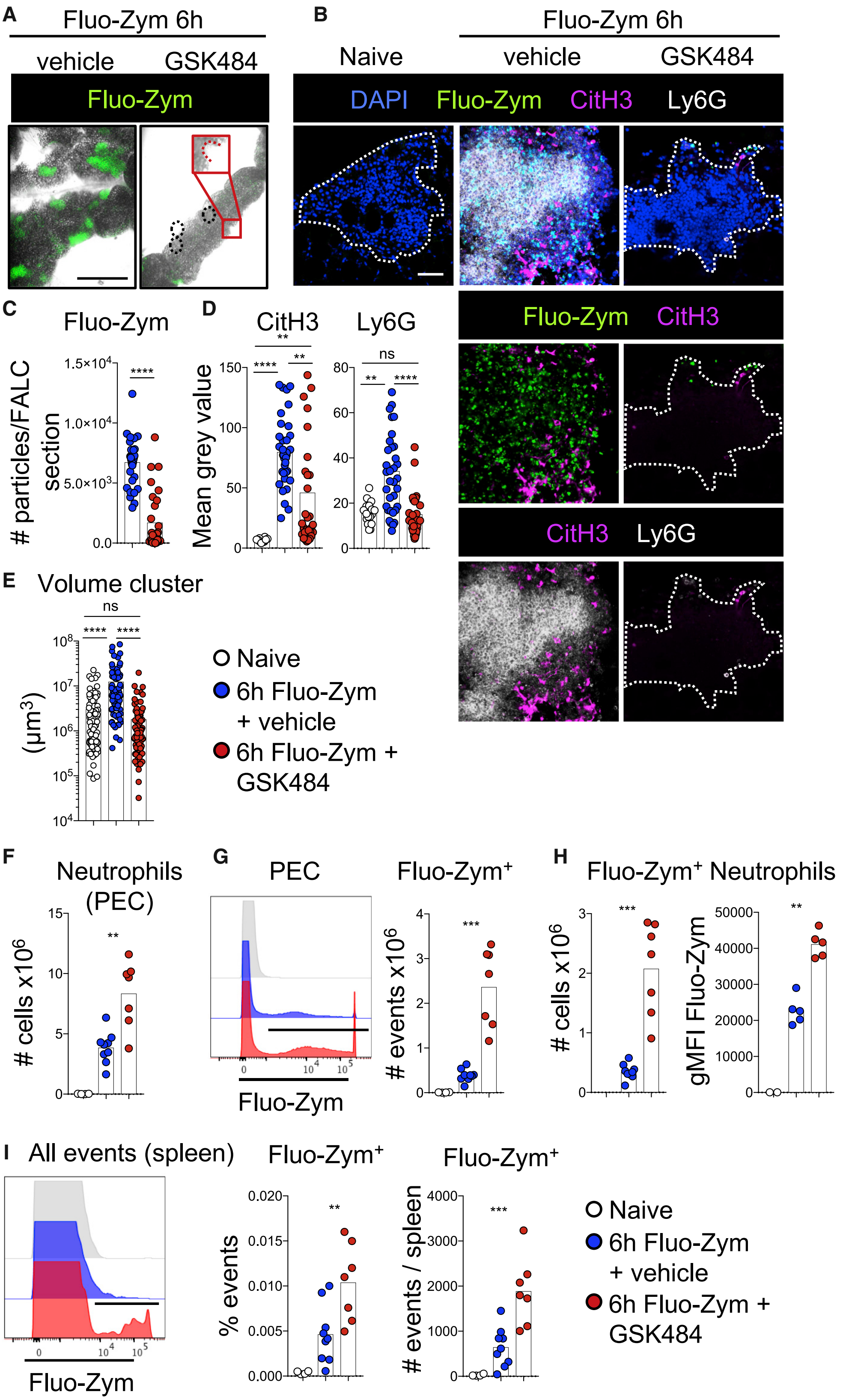

(legend on next page)

FALCs between 6 and 18 h post-Zymosan injection at the peak of neutrophil recruitment (Figures 4B, 3A, and 3C). The highest density of CitH3$^+$ DNA was found on the surface of the expanded FALCs, which typically formed a dense core of neutrophils coated with a CitH3$^+$ DNA outer-layer (Figures 4C, S6A, and S6B).

To further characterize the CitH3$^+$ DNA outer-layer and determine if it could be considered as NETs, we analyzed the requirement for Neutrophil elastase (NE). NE is a granule serine protease which translocates to the nucleus, where it promotes chromatin decondensation and the formation of NETs (Papayannopoulos et al., 2010). Here, we found that the formation of the CitH3$^+$ DNA layer coating omFALC neutrophil aggregates, and the expansion of these structures during Zymosan-induced peritonitis was not affected in NE-deficient *Elane*$^{-/-}$ mice compared to wild-type (WT) mice (Figure 4D). MPO, another granule serine protease, synergizes the action of NE and is found associated with NETs (Papayannopoulos et al., 2010). Staining for MPO revealed that the CitH3$^+$ DNA covering the neutrophil aggregates was not associated with MPO, suggesting that MPO relocation to the nucleus is not required for the formation of the CitH3$^+$ DNA outer-layer (Figure 4E). Given that the neutrophil aggregates formed independently of NE, we sought to confirm that the aggregates were formed by neutrophils. Injection of anti-Gr1 antibodies successfully mediated depletion of the Ly6G$^+$ peritoneal neutrophil population at 6 h following Zymosan-A intraperitoneal (i.p.) injection (Figure S7A). Following depletion of peritoneal neutrophils, no accretion of Ly6G$^+$ neutrophils, staining for CitH3$^+$, nor expansion in cluster volume occurred within the omentum (Figure S7B). Therefore, the formation of the CitH3$^+$ DNA layer coating the omFALC aggregates that appear during Zymosan-induced peritonitis is formed by neutrophils independently of NE and thus different from the formation of "classical" NETs.

Inhibition of PAD4 Prevents the Aggregation of Neutrophils in omFALCs and the Trapping of Zymosan by omFALCs

The enzyme PAD4, which mediates the conversion of arginine into citrulline, is implicated *in vitro* and *in vivo* in the formation of NETs. The role of PAD4 in NET formation remains controversial and seems to be context dependent (Boeltz et al., 2019; Konig and Andrade, 2016). In order to test whether PAD4 was involved in the capture of particulate contaminants and the formation of neutrophil aggregates on omFALCs, we used fluorescently labeled Zymosan (Fluo-Zym) and the specific PAD4 inhibitor GSK484 (Lewis et al., 2015). Observation of the omentum with a stereo-microscope at 6 h post-injection revealed that omFALCs had undergone a massive expansion and very effectively captured and concentrated Fluo-Zym particles in FALCs (Figure 5A). In contrast to the omentum, the epidydimal fat pad, parietal wall, and diaphragm did not capture any Fluo-Zym (Figure S7C). The Fluo-Zym particles were concentrated in dispersed spots on the mesenteries in keeping with the lower proportion of FALCs within this tissue compared to the omentum. PAD4 inhibition completely abrogated both the omFALC expansion and the capture of Fluo-Zym particles by omFALCs (Figures 5A–5C). Fluo-Zym injection led to the formation of large neutrophil aggregates embedded in CitH3$^+$ DNA, while PAD4 inhibition blocked the recruitment of Ly6G$^+$ neutrophils into omFALCs and significantly reduced the CitH3 staining compared to Fluo-Zym-only controls (Figures 5B and 5D). PAD4 inhibition was associated with omFALCs failing to capture Fluo-Zym particles and expand in size (Figures 5A and 5E).

In contrast, GSK484 did not block the recruitment of neutrophils into the peritoneal cavity since we recovered twice as many neutrophils from the peritoneal cavity when mice received GSK484 and Fluo-Zym compared to Fluo-Zym only (Figure 5F). Thus, GSK484 inhibits neutrophil accretion in omFALCs, leading to a very severe reduction in the capacity of the omentum to capture Zymosan particles. While neutrophils clearly underwent citrullination of Histone H3 during Zymosan-induced peritonitis, the fact that PAD4 inhibition led to a near-complete abrogation of neutrophil aggregation within FALCs did not allow us to conclude whether PAD4 was involved in the formation of the CitH3$^+$ DNA outer-layer we observed.

Inhibition of PAD4 Leads to Impaired Clearance of Zymosan Particles from the Peritoneal Cavity and Increases Dissemination to the Spleen

We next addressed the effect of PAD4 inhibition on peritoneal neutrophil clearance of Zymosan-A. Inhibition of PAD4 resulted in a significant increase in the retention of neutrophils and Fluo-Zym particles within the peritoneal cavity (Figures 5F and 5G). There was a 3-fold increase in the proportion of neutrophils retaining Fluo-Zym particles, with a near 2-fold increase in Fluo-Zym mean fluorescence intensity (MFI), indicating that PAD4 inhibition led to increased phagocytosis of Fluo-Zym particles by peritoneal neutrophils (Figure 5H). Finally, PAD4 inhibition led to increased dissemination of Fluo-Zym particles to the spleen (Figure 5I). Thus, this suggests that PAD4-dependent neutrophil aggregation within omFALCs provides a rapid and efficient

Figure 5. Inhibition of PAD4 Prevents Neutrophil Aggregation and Capture of Zymosan Particles within omFALCs While Increasing the Retention of Zymosan in the Peritoneal Cavity and Spread to the Spleen

Mice were left naive (white) or injected i.p. with Fluo-Zym in combination with the PAD4 inhibitor GSK484 (red) or vehicle (blue) and the omentum. PECs and spleen were analyzed 6 h post injection.

(A and B) Representative low (A, scale bar 1 mm) and high (B, scale bar 50 μm) magnification confocal images of wholemount immunofluorescence staining of omentum.

(C–E) Number of Fluo-Zym particles per mm^2 of omFALCs (C), mean gray value for CitH3 and Ly6G staining (D), volume of omFALCs (E).

(F) Total number of PEC neutrophils.

(G) Representative histogram showing fluorescence intensity of Fluo-Zym in all PECs and number of Fluo-Zym$^+$ PECs.

(H) Number of Fluo-Zym$^+$ neutrophils and MFI of Fluo-Zym within Fluo-Zym$^+$ neutrophils.

(I) Representative histogram showing fluorescence intensity of Fluo-Zym in spleen and percentage and number of Fluo-Zym$^+$ events per spleen.

Data for cluster quantification pooled from two independent experiments with $n \geq 30$ (C–D) and $n \geq 60$ (E) imaged clusters from n = 7–8 mice per group. Data pooled from two independent experiments with n = 7–8 mice per group. Kruskal Wallis test with Dunn's multiple comparisons test or Mann Whitney test was applied after assessing normality using D'Agostino and Pearson normality test, ns = non-significant, ** = $p < 0.01$, ***p = < 0.001, ****p = < 0.0001.

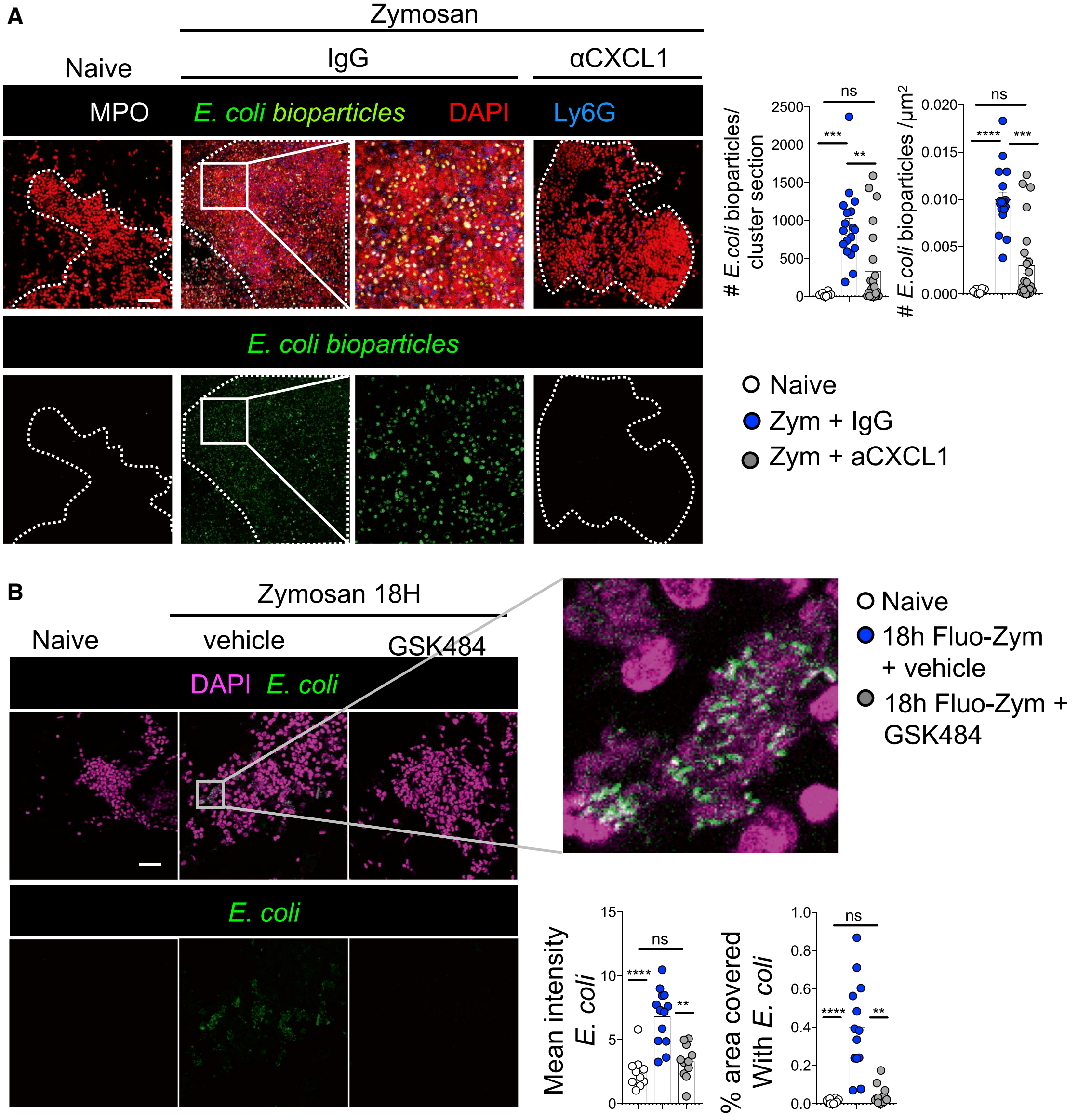

Figure 6. CXCL1 and PAD4 Are Required for the Adhesion of Bacteria to the Omentum in the Absence of Fluid Flow

(A) Representative confocal images of wholemount staining of omenta, isolated from either naive animals or 18 h after Zymosan i.p injection, in combination with the injection of either anti-CXCL1 or isotype control antibodies, and cultured with fluorescently labeled *E. coli* bio-particles for 10 min. Number of *E. coli* bio-particles found per cluster section and per μm^2 of cluster were graphed. MPO (white), *E. coli* (green), DAPI (red), Ly6G (blue). Data for cluster quantification pooled from two independent experiments with $n \geq 18$ imaged clusters from n = 10–11 mice per group. Error bars show SEM.

(B) Mice were left naive (white circles) or injected i.p. with Zymosan in combination with the PAD4 inhibitor GSK484 (gray circles) or vehicle (blue circles). The omenta were collected 18 h post-injection and incubated *in vitro* for 5 min with mCherry^{+} *E. coli*. Representative confocal images of wholemount staining of omentum. *E. coli* (green), DAPI (magenta). Mean gray value of *E. coli* in omFALCs and percentage area covered by *E. coli.*

Data for cluster quantification pooled from two independent experiments with $n \geq 10$ imaged clusters from n = 7–8 mice per group. Error bars show SEM. Kruskal Wallis test with Dunn's multiple comparisons test were applied after assessing normality using D'Agostino and Pearson normality tests, ns = non-significant, ** = $p < 0.01$, ***p = < 0.001, ****p = < 0.0001. Scale bars, 50 μm.

mechanism to clear particulate material from the peritoneal cavity and limit the systemic spread of peritoneal contaminants.

Neutrophil Recruitment and Aggregation Confer Increased Adhesive Properties to omFALCs

In the peritoneal cavity, the capture of particles by omFALCs results from a combination of peritoneal fluid flow through the omentum and the capacity of the omentum to retain these particles. To assess the importance of neutrophil aggregation on the adhesive properties of the omentum independent of fluid flow, we measured *ex vivo* the capacity of the omentum to capture bacteria relevant to peritonitis by briefly incubating fluorescently labeled *E. coli* bioparticles with omentum samples isolated from untreated control mice, or from

mice undergoing Zymosan-induced peritonitis that had been treated with isotype control antibodies or anti-CXCL1 antibodies *in vivo* (Figure 6A). Omenta were collected at 18 h post-Zymosan injection and incubated *ex vivo* for 5 min at room temperature with *E. coli* bioparticles before fixation and wholemount immunofluorescence imaging. Omentum tissue sampled from mice with peritonitis proficiently trapped *E. coli*, whereas those from control mice without peritonitis did not (Figure 6A). Blockade of CXCL1 resulted in fewer neutrophils within the omentum and a failure to efficiently trap *E. coli,* indicating that CXCL1-mediated recruitment of neutrophils to the omentum dramatically increased the capacity of the omentum to capture bacterial contaminants. We confirmed the importance of neutrophil recruitment and aggregation for the increased adhesive properties of the omentum during peritonitis using omenta isolated from naive mice, those undergoing peritonitis, those undergoing peritonitis with PAD4 inhibition, and *E. coli* expressing the fluorescent protein mCherry. Omenta from naive mice did not trap *E. coli*, contrary to omenta from mice with peritonitis (containing NET-like structures), where large areas of FALCs were covered in *E. coli.* PAD4 inhibition led to a marked decrease in the efficiency with which the omentum captured *E. coli* (Figure 6B). These data suggest that neutrophil aggregation within omFALCs during peritonitis contributes to omental clearance of peritoneal bacterial contaminants.

Neutrophils Are Recruited to the Human Omentum during Peritonitis

We reasoned that appendicitis would provide a useful translational platform to examine the function of the omentum during peritonitis. During acute appendicitis, the omentum wraps itself around and adheres to the inflamed appendix (Morison, 1906). By comparison, in patients undergoing laparoscopic surgery for biliary colic (surgery for gallstones without active inflammation), the omentum and appendix are both uninvolved. We recruited patients who were undergoing laparoscopic surgery for possible or suspected acute appendicitis or laparoscopic cholecystectomy for biliary colic (non-inflamed, NI; Table S2). Acute appendicitis induced a stark influx of $CD16^{+}CD14^{-}CD19^{-}CD3^{-}CD56^{-}CD15^{+}$ neutrophils into the omentum and the peritoneal cavity (Figures 7A and 7B).

We next investigated whether the mechanisms regulating recruitment of neutrophils to the human omentum were similar to mouse by analyzing the secretion of inflammatory chemokines and cytokines over a 2 h interval in *ex vivo* human omentum explant cultures (Figure 7C). CXCL8 (human homolog of murine CXCL1) and IL-1β, two important factors for neutrophil recruitment and activation (Biondo et al., 2014), were secreted at much higher levels in omental explant cultures and peritoneal lavage in inflammatory conditions compared to controls, but not in serum (Figures 7C and 7D). In patients with acute appendicitis, 1 g of omental explant released 25 ng of CXCL8, approximately 400 times more than the amount found per ml of peritoneal lavage, despite the number of neutrophils being similar in equivalent volumes of omentum and wash fluid (Figures 7B and 7D), indicating that the human omentum is a key site of CXCL8 release and neutrophil recruitment during peritonitis.

$CitH3^{+}$ NET-like Structures Are Released on the Human Omentum during Peritonitis

Finally, we tested whether neutrophils recruited to the omentum during peritonitis in humans also released $CitH3^{+}$ NET-like structures. Wholemount immunofluorescence staining showed the presence of extracellular DNA fibrils stained with SYTOX or DAPI, which co-localize with CitH3, in regions where there was a substantial $CD15^{+}$ neutrophil infiltrate during acute appendicitis (Figure 7E). Areas of $CitH3^{+}$ DNA staining which did not co-localize with MPO were found, comparable to the murine omentum during peritonitis. No NET-like structures were detected in any NI control samples (Figure 7E). Double-stranded (ds)DNA was found in omental explant culture supernatants and in the peritoneal wash fluid from patients with acute appendicitis, but not from control patients (Figure 7F). Since an equivalent number of neutrophils were present per g of omentum and per ml of peritoneal wash (Figure 7B), the fact that dsDNA was released in the greatest amounts by the omental explants strongly suggests that the omentum supports DNA release from neutrophils. Using a limulus amoebocyte lysate (LAL) assay, we measured bacterial LPS within omental explant cultures and detected LPS release into culture supernatants from omentum isolated from patients with acute appendicitis, but not from NI patients. Patients with appendicitis also had higher serum levels of LPS, but there was no evidence of LPS within the peritoneal wash (Figure 7G). This suggested that during appendicitis, neutrophil aggregation and the release of $CitH3^{+}$ NET-like structures on the omentum mediate successful capture of bacterial antigens arising from the inflamed appendix and thus protect the wider peritoneal cavity from contamination and generalized peritonitis.

DISCUSSION

In the present study, we uncovered important facets of stromal-immune cell interactions, which governed omFALC function within the peritoneal cavity and revealed how neutrophils mediated the clearance of peritoneal contaminants by omFALCs. The surface of FALCs was covered by differentiated mesothelial cells specialized in the secretion of inflammatory mediators (*Cxcl13*$^{+}$ FALC cover cells) and the response to virus (*Ifit*$^{+}$ FALC cover cells). During peritonitis, neutrophils rapidly accumulated within omFALCs, where they formed large aggregates concentrating peritoneal contaminants. The formation of these aggregates was dependent on two mechanisms: (1) CXCL1, which was produced by $Cxcl13^{+}$ FALC cover cells; and (2) the PAD4-dependent formation of a $CitH3^{+}$ DNA outer-layer coating the omFALC neutrophil aggregates. In humans with appendicitis, the omentum was also a site of neutrophil recruitment and the release of $CitH3^{+}$ DNA. In addition, we provided evidence that the omentum efficiently captured bacterial antigens, leaving the peritoneal cavity free of contamination.

In the omentum, the mesothelium acts as a filtration system for peritoneal fluid. Entrance of peritoneal fluid is facilitated by the presence of stomata (Mironov et al., 1979) that afford particles and cells entrance into FALCs and enable fluid to be drained into neighboring lymphatic vessels. Our analysis revealed the exquisite adaptation of the mesothelium for this function, which gave rise to *Cxcl13*$^{+}$ FALC cover cells, specialized in the

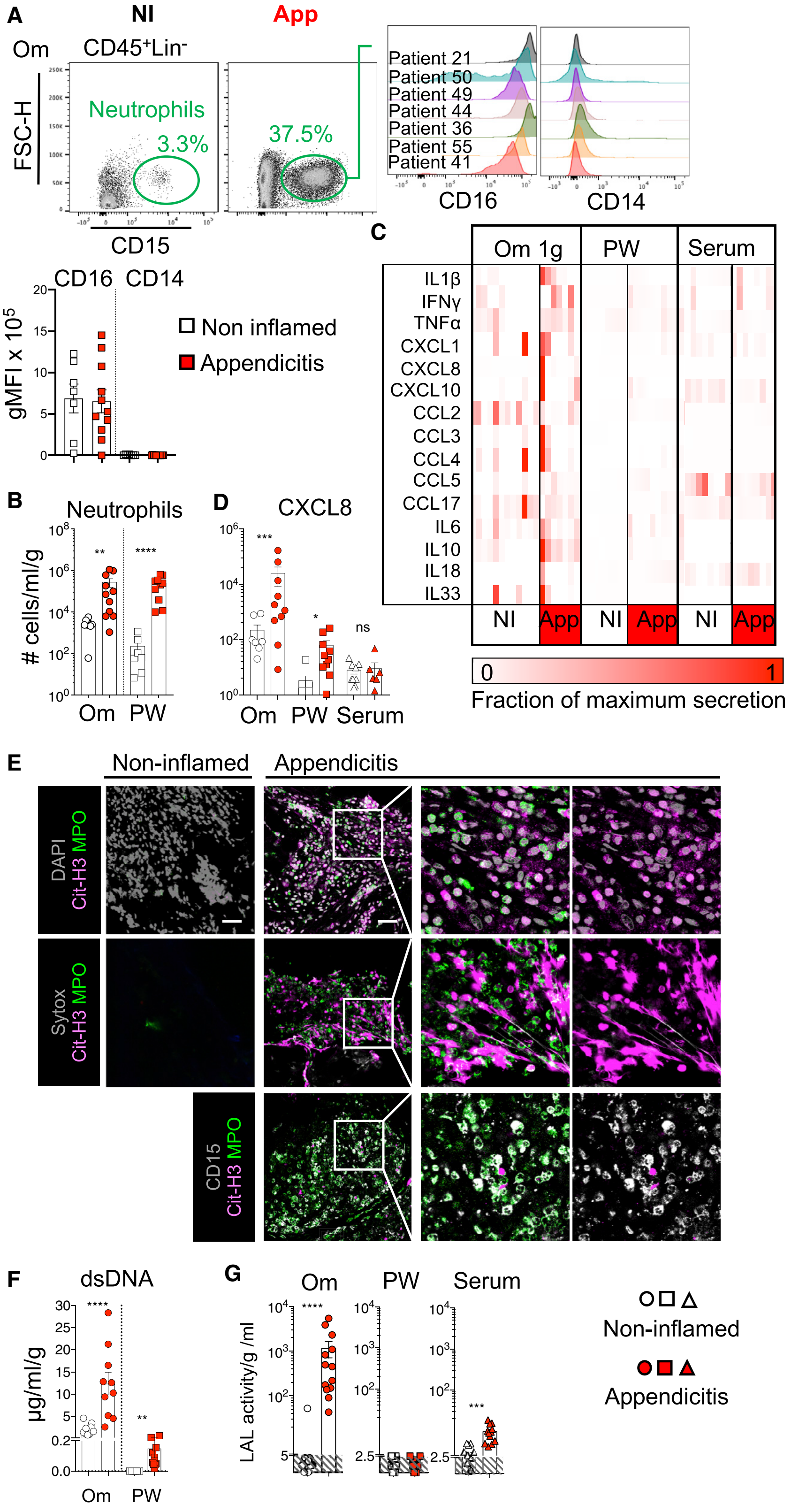

Figure 7. The Human Omentum Recruits Neutrophils and Collects Bacterial Antigens during Appendicitis

(A,) Representative gating strategy showing CD45^{+} CD19^{-}, CD3^{-}, NCAM^{-}, and CD15^{+} neutrophils in the omentum (Om) of non-inflamed (NI) and appendicitis (App) patients. Histogram showing expression of CD16 and CD14 by omental neutrophils from representative patients with App, and quantification of CD16 and CD14 MFI on CD15^{+} neutrophils from NI (white) and appendicitis (red) patients.

(B) Number of neutrophils found per g of Om or per ml of peritoneal wash (PW) of NI and App patients. Patients were stratified based on surgical outcome into one of two groups, NI (white) or App (red), n = 7 and 10 patients per group. Error bars show SEM.

(C,) Chemokines and cytokines found in 2 h Om explant culture supernatant, PW, and serum comparing NI and App patients. Each column represents one patient.

(D) Amounts of CXCL8 per g per ml of 2 h Om explant culture supernatant.

(E) Representative confocal images of wholemount immunofluorescence staining of omentum biopsies from NI or App patients (n $\geq$ 6) showing in gray DNA stained with DAPI (upper) or extracellular DNA stained with SYTOX (middle, gray), or CD15 (lower); in magenta CD11b; in green MPO and in red Citrullinated histone H3 (CitH3).

(F) Amounts of double-stranded DNA (dsDNA) released into the supernatant of 2 h omental explant culture per g and per ml and per ml of PW in control and App patients.

(G) LAL activity within the supernatant of 2 h omental explant culture per g and per ml of PW and serum in control and appendicitis patients. n = 10–13 patients per group.

Error bars show SEM. Unpaired Student's t test or Mann Whitney test was applied after assessing normality using D'Agostino and Pearson and Shapiro-Wilk normality tests, ns = non-significant, * = $p < 0.05$, ** = $p < 0.01$, ***p = < 0.001.

attraction of immune cells and the secretion of inflammatory mediators and *Ifit*$^+$ FALC cover cells competent for the secretion of anti-viral factors. Cxcl1 was expressed by both *Cxcl13*$^+$ FALC stromal cells and *Pdgfra*$^+$ fibroblast, but only FALC cover cells had intra-cellular stores of CXCL1, suggesting that CXCL1 production was post-transcriptionally regulated in FALC cover cells. In absence of a genetic model allowing the specific deletion of *Cxcl1* in FALC cover cells, we cannot rule out that CXCL1 from other cellular sources is important for the recruitment of neutrophils to the omentum. Based on our results, we hypothesize that following the initial *Cxcl13*$^+$ FALC cover cell CXCL1 mediated recruitment of zymosan-loaded peritoneal neutrophils to omFALCs; the release of extracellular DNA facilitates the adhesion of further waves of neutrophils, which also extrude their DNA, resulting in the large aggregates imaged at 6–18 h. To account for the staining pattern seen, we assume that re-modeling of the DNA extrusions must occur to clear CitH3 from the center of the aggregates while leaving the fluid-facing aggregates coated in NET-like structures. This intriguing CitH3$^+$ DNA outer-coating raises multiple questions., (1) what is the role of the CitH3$^+$ DNA on the fluid facing surface of the cluster; are CitH3$^+$ neutrophil-aggregated omFALCs anti-bacterial? (2) Are the aggregations performing a purely structural role by barricading the omFALC to limit the spread of peritoneal contaminants? (3) How are the aggregates cleared during the resolution of peritonitis? (4) Does resolution require exposure of CitH3$^+$ DNA?

In sepsis, NETs promote a toxic inflammatory and procoagulant host response to endotoxin (Martinod et al., 2015). However, our data point toward an important role for omental NET release to limit the propagation of contaminants from the peritoneal cavity to the circulation. Due to constant drainage of peritoneal fluid through FALCs and their high vascularization (Buscher et al., 2016; Dickinson, 1906; Gray et al., 2012), FALCs are the main portals for the systemic transit of peritoneal contaminants. Additional experiments are required to further investigate the effect of PAD4 inhibition on neutrophil trafficking to secondary lymphoid organs during peritonitis; altered neutrophil trafficking may influence the export of peritoneal contaminants through the draining LNs and spleen. While targeted NET release on omFALCs is beneficial during peritonitis, it may play a detrimental role in ovarian cancer metastasis (Lee et al., 2019).

Resident peritoneal macrophages undergo a clotting response within the first 2 h of contamination of the peritoneal cavity, providing a means of clearing peritoneal contaminants via coagulation and adhesion (Zhang et al., 2019). This mechanism serves to convert fluid phase inflammation to a solid state within the clots. Neutrophil aggregation within FALCs also enables the conversion to a solid state, which is required for efficient clearing of particles from the fluid phase. In doing so, neutrophils provide a timely relay for the neutralization of peritoneal contaminants, since resident peritoneal macrophages are rapidly sequestered in clots (Zhang et al., 2019). In gout and pancreatitis, presence of high-density neutrophils in combination with the release of NETs leads to the formation of large DNA aggregates. In gout, these aggregated NETs have the ability to degrade cytokines and chemokines via serine proteases and may be important to limit inflammation (Schauer et al., 2014). In pancreatitis, PAD4 mediates the release of NETs, which cause neutrophil aggregation and occlusion of pancreatic ducts (Leppkes et al., 2016). Aggregated NETs have been implicated in the formation of gallstones (Muñoz et al., 2019); Muñoz et al. report that uptake of small crystals results in NET release that is dependent upon PAD4. Here, we show that omFALCs are also sites where high densities of actively phagocytosing neutrophils aggregate in a PAD4-dependent mechanism during peritonitis.

Previous studies have posited that both MPO and NE are necessary for the release of bona fide NETs (Metzler et al., 2011; Papayannopoulos et al., 2010). Here, we found that the release of CitH3$^+$ DNA and aggregation of neutrophils over the surface of expanded omFALCs during peritonitis occurred even in the absence of NE, suggesting a mechanism of DNA release that did not conform to the same rules as classical NET formation. In addition, CitH3$^+$ DNA did not robustly co-localize with MPO staining, again indicating a non-classical mechanism of neutrophil CitH3$^+$ DNA release. In contrast to NE, the formation of omFALC neutrophil aggregates was dependent upon PAD4. Taken together, our findings contribute to mounting evidence that mechanisms of NET release vary, dependent upon context and location (Boeltz et al., 2019).

STAR★METHODS

Detailed methods are provided in the online version of this paper and include the following:

- KEY RESOURCES TABLE
- RESOURCE AVAILABILITY
  - Lead Contact
  - Materials availability
  - Data and Code Availability
- EXPERIMENTAL MODEL AND SUBJECT DETAILS
  - Experimental animals
  - Human subjects
- METHOD DETAILS
  - Peritonitis models
  - Human sample preparation
  - Flow cytometry
  - Cell-sorting and quantitative real-time PCR
  - Detection of chemokines, cytokine, dDNA and LPS
  - Immunofluorescence staining and microscopy
  - Droplet based scRNaseq and data pre-processing
  - scRNaseq data analysis and visualization
  - Mesothelium trajectory inference
  - Mapping mesenteric single cell data
  - RNaseq data analysis
- QUANTIFICATION AND STATISTICAL ANALYSIS

SUPPLEMENTAL INFORMATION

Supplemental Information can be found online at https://doi.org/10.1016/j.immuni.2020.03.011.

ACKNOWLEDGMENTS

We are grateful to the Edinburgh Emergency Surgery Study Group, Royal Infirmary of Edinburgh, NHS Lothian: Graeme Couper, Christopher Deans, Gavin G.P. Browning, Anna M Paisley, Bruce Tulloh, Richard J.E. Skipworth, Simon Paterson-Brown, Rajan Ravindran, Andrew de Beaux, Stuart McKechnie,

Andrew Healey, Dimitri Damaskos, Marina Garcia, and all Royal Infirmary surgical trainees for facilitating intraoperative sample collection. We thank the Wellcome Trust Clinical Research Facility, NHS Lothian, especially Beena Poulose, for clinical study support. Flow cytometry and confocal microscopy data were generated with support from the QMRI flow-cytometry and cell-sorting facility, the CALM facility, and CRM-Imaging, University of Edinburgh. Stephen Mitchell provided assistance with SEM. Nadin Fathallah provided technical assistance. We thank Central Bioresearch Services, especially Gidona Goodman, Chris Flockhart, and Will Mungall for their technical support; and D. Leach (University of Edinburgh) for the RecA-mCherry *E. coli*. The graphical abstract was compiled using images from Servier Medical Art. This work was supported by a Medical Research Council (MRC) UK grant to C.B. (MR/M011542/1) and S.V. (MR/S008020/1), a British Heart Foundation Centre for Research Excellence award III (RE/18/5/34216), and a Wellcome Trust Seed award in Science to L.H.J.-J. (213697/Z/18/Z). D.J.M. was funded by an MRC UK Senior Clinical Fellowship (MR/P008887/1) and N.C.H. was supported by a Wellcome Trust Senior Research Fellowship in Clinical Science (ref. 103749).

AUTHOR CONTRIBUTIONS

L.H.J.-J: Conceptualization, Investigation, Supervision, Project Administration, Formal Analysis, Writing – Original Draft, and Visualization. P.S., M.S.M., K.J.M., J.R.P., N.C.H., L.D., and S.V.: Investigation, Analysis, and Review. B.E.P.H., R.D., M.N., and M.M.V.: Investigation. D.J.M.: Clinical Protocol Draft, Obtaining Ethical and Clinical Governance permissions, Chief Investigator (Clinical Arm), Methodology, Project Administration, and Writing – Review & Editing. C.B.: Funding Acquisition, Supervision, Conceptualization, Investigation, Project Administration, Formal Analysis, Writing – Original Draft, and Visualization.

DECLARATION OF INTERESTS

The authors declare no competing interests.

Received: September 26, 2019
Revised: February 11, 2020
Accepted: March 18, 2020
Published: April 14, 2020

REFERENCES

Amarh, V., White, M.A., and Leach, D.R.F. (2018). Dynamics of RecA-mediated repair of replication-dependent DNA breaks. J. Cell Biol. *217*, 2299–2307.

Ansel, K.M., Harris, R.B., and Cyster, J.G. (2002). CXCL13 is required for B1 cell homing, natural antibody production, and body cavity immunity. Immunity *16*, 67–76.

Belaaouaj, A., McCarthy, R., Baumann, M., Gao, Z., Ley, T.J., Abraham, S.N., and Shapiro, S.D. (1998). Mice lacking neutrophil elastase reveal impaired host defense against gram negative bacterial sepsis. Nat. Med. *4*, 615–618.

Bénézech, C., Luu, N.T., Walker, J.A., Kruglov, A.A., Loo, Y., Nakamura, K., Zhang, Y., Nayar, S., Jones, L.H., Flores-Langarica, A., et al. (2015). Inflammation-induced formation of fat-associated lymphoid clusters. Nat. Immunol. *16*, 819–828.

Biondo, C., Mancuso, G., Midiri, A., Signorino, G., Domina, M., Lanza Cariccio, V., Mohammadi, N., Venza, M., Venza, I., Teti, G., and Beninati, C. (2014). The interleukin-1β/CXCL1/2/neutrophil axis mediates host protection against group B streptococcal infection. Infect. Immun. *82*, 4508–4517.

Blighe, K.R.S., and Lewis, M. (2019). EnhancedVolcano: Publication-ready volcano plots with enhanced colouring and labeling. R package version 1.4.0 https://github.com/kevinblighe/EnhancedVolcano.

Boeltz, S., Amini, P., Anders, H.J., Andrade, F., Bilyy, R., Chatfield, S., Cichon, I., Clancy, D.M., Desai, J., Dumych, T., et al. (2019). To NET or not to NET:current opinions and state of the science regarding the formation of neutrophil extracellular traps. Cell Death Differ. *26*, 395–408.

Buechler, M.B., Kim, K.W., Onufer, E.J., Williams, J.W., Little, C.C., Dominguez, C.X., Li, Q., Sandoval, W., Cooper, J.E., Harris, C.A., et al. (2019). A Stromal Niche Defined by Expression of the Transcription Factor WT1 Mediates Programming and Homeostasis of Cavity-Resident Macrophages. Immunity *51*, 119–130.e5.

Buscher, K., Wang, H., Zhang, X., Striewski, P., Wirth, B., Saggu, G., Lütke-Enking, S., Mayadas, T.N., Ley, K., Sorokin, L., and Song, J. (2016). Protection from septic peritonitis by rapid neutrophil recruitment through omental high endothelial venules. Nat. Commun. *7*, 10828.

Chau, Y.Y., Bandiera, R., Serrels, A., Martínez-Estrada, O.M., Qing, W., Lee, M., Slight, J., Thornburn, A., Berry, R., McHaffie, S., et al. (2014). Visceral and subcutaneous fat have different origins and evidence supports a mesothelial source. Nat. Cell Biol. *16*, 367–375.

Croft, A.P., Campos, J., Jansen, K., Turner, J.D., Marshall, J., Attar, M., Savary, L., Wehmeyer, C., Naylor, A.J., Kemble, S., et al. (2019). Distinct fibroblast subsets drive inflammation and damage in arthritis. Nature *570*, 246–251.

Cruz-Migoni, S., and Caamaño, J. (2016). Fat-Associated Lymphoid Clusters in Inflammation and Immunity. Front. Immunol. *7*, 612.

Dickinson, G.K. (1906). II. The Omentum and its Functions. Ann. Surg. *44*, 652–665.

Doherty, N.S., Griffiths, R.J., Hakkinen, J.P., Scampoli, D.N., and Milici, A.J. (1995). Post-capillary venules in the "milky spots" of the greater omentum are the major site of plasma protein and leukocyte extravasation in rodent models of peritonitis. Inflamm. Res. *44*, 169–177.

Fabregat, A., Jupe, S., Matthews, L., Sidiropoulos, K., Gillespie, M., Garapati, P., Haw, R., Jassal, B., Korninger, F., May, B., et al. (2018). The Reactome Pathway Knowledgebase. Nucleic Acids Res. *46* (D1), D649–D655.

Gray, K.S., Collins, C.M., and Speck, S.H. (2012). Characterization of omental immune aggregates during establishment of a latent gammaherpesvirus infection. PLoS ONE *7*, e43196.

Hodel, C. (1970). Ultrastructural studies on the absorption of protein markers by the greater omentum. Eur. Surg. Res. *2*, 435–449.

Huang, H.Y., Rivas-Caicedo, A., Renevey, F., Cannelle, H., Peranzoni, E., Scarpellino, L., Hardie, D.L., Pommier, A., Schaeuble, K., Favre, S., et al. (2018). Identification of a new subset of lymph node stromal cells involved in regulating plasma cell homeostasis. Proc. Natl. Acad. Sci. USA *115*, E6826–E6835.

Jackson-Jones, L.H., and Bénézech, C. (2018). Control of innate-like B cell location for compartmentalised IgM production. Curr. Opin. Immunol. *50*, 9–13.

Jackson-Jones, L.H., Duncan, S.M., Magalhaes, M.S., Campbell, S.M., Maizels, R.M., McSorley, H.J., Allen, J.E., and Bénézech, C. (2016). Fat-associated lymphoid clusters control local IgM secretion during pleural infection and lung inflammation. Nat. Commun. *7*, 12651.

Jin, L., Batra, S., and Jeyaseelan, S. (2017). Diminished neutrophil extracellular trap (NET) formation is a novel innate immune deficiency induced by acute ethanol exposure in polymicrobial sepsis, which can be rescued by CXCL1. PLoS Pathog. *13*, e1006637.

Kiselev, V.Y., Yiu, A., and Hemberg, M. (2018). scmap: projection of single-cell RNA-seq data across data sets. Nat. Methods *15*, 359–362.

Koga, S., Hozumi, K., Hirano, K.I., Yazawa, M., Terooatea, T., Minoda, A., Nagasawa, T., Koyasu, S., and Moro, K. (2018). Peripheral PDGFRα^{+}gp38^{+} mesenchymal cells support the differentiation of fetal liver-derived ILC2. J. Exp. Med. *215*, 1609–1626.

Konig, M.F., and Andrade, F. (2016). A Critical Reappraisal of Neutrophil Extracellular Traps and NETosis Mimics Based on Differential Requirements for Protein Citrullination. Front. Immunol. *7*, 461.

Lee, W., Ko, S.Y., Mohamed, M.S., Kenny, H.A., Lengyel, E., and Naora, H. (2019). Neutrophils facilitate ovarian cancer premetastatic niche formation in the omentum. J. Exp. Med. *216*, 176–194.

Leppkes, M., Maueröder, C., Hirth, S., Nowecki, S., Günther, C., Billmeier, U., Paulus, S., Biermann, M., Munoz, L.E., Hoffmann, M., et al. (2016). Externalized decondensed neutrophil chromatin occludes pancreatic ducts and drives pancreatitis. Nat. Commun. *7*, 10973.

Lewis, H.D., Liddle, J., Coote, J.E., Atkinson, S.J., Barker, M.D., Bax, B.D., Bicker, K.L., Bingham, R.P., Campbell, M., Chen, Y.H., et al. (2015).

Inhibition of PAD4 activity is sufficient to disrupt mouse and human NET formation. Nat. Chem. Biol. *11*, 189–191.

Love, M.I., Huber, W., and Anders, S. (2014). Moderated estimation of fold change and dispersion for RNA-seq data with DESeq2. Genome Biol. *15*, 550.

Mahlakõiv, T., Flamar, A.L., Johnston, L.K., Moriyama, S., Putzel, G.G., Bryce, P.J., and Artis, D. (2019). Stromal cells maintain immune cell homeostasis in adipose tissue via production of interleukin-33. Sci. Immunol. *4*, https://doi.org/10.1126/sciimmunol.aax0416.

Malhotra, D., Fletcher, A.L., Astarita, J., Lukacs-Kornek, V., Tayalia, P., Gonzalez, S.F., Elpek, K.G., Chang, S.K., Knoblich, K., Hemler, M.E., et al.; Immunological Genome Project Consortium (2012). Transcriptional profiling of stroma from inflamed and resting lymph nodes defines immunological hallmarks. Nat. Immunol. *13*, 499–510.

Martinod, K., Fuchs, T.A., Zitomersky, N.L., Wong, S.L., Demers, M., Gallant, M., Wang, Y., and Wagner, D.D. (2015). PAD4-deficiency does not affect bacteremia in polymicrobial sepsis and ameliorates endotoxemic shock. Blood *125*, 1948–1956.

Metzler, K.D., Fuchs, T.A., Nauseef, W.M., Reumaux, D., Roesler, J., Schulze, I., Wahn, V., Papayannopoulos, V., and Zychlinsky, A. (2011). Myeloperoxidase is required for neutrophil extracellular trap formation: implications for innate immunity. Blood *117*, 953–959.

Meza-Perez, S., and Randall, T.D. (2017). Immunological Functions of the Omentum. Trends Immunol. *38*, 526–536.

Mironov, V.A., Gusev, S.A., and Baradi, A.F. (1979). Mesothelial stomata overlying omental milky spots: scanning electron microscopic study. Cell Tissue Res. *201*, 327–330.

Morison, R. (1906). Remarks ON SOME FUNCTIONS OF THE OMENTUM. BMJ *1*, 76–78.

Moro, K., Yamada, T., Tanabe, M., Takeuchi, T., Ikawa, T., Kawamoto, H., Furusawa, J., Ohtani, M., Fujii, H., and Koyasu, S. (2010). Innate production of T(H)2 cytokines by adipose tissue-associated c-Kit(+)Sca-1(+) lymphoid cells. Nature *463*, 540–544.

Muñoz, L.E., Boeltz, S., Bilyy, R., Schauer, C., Mahajan, A., Widulin, N., Grüneboom, A., Herrmann, I., Boada, E., Rauh, M., et al. (2019). Neutrophil Extracellular Traps Initiate Gallstone Formation. Immunity *51*, 443–450.e4.

Papayannopoulos, V. (2018). Neutrophil extracellular traps in immunity and disease. Nat. Rev. Immunol. *18*, 134–147.

Papayannopoulos, V., Metzler, K.D., Hakkim, A., and Zychlinsky, A. (2010). Neutrophil elastase and myeloperoxidase regulate the formation of neutrophil extracellular traps. J. Cell Biol. *191*, 677–691.

Patro, R., Duggal, G., Love, M.I., Irizarry, R.A., and Kingsford, C. (2017). Salmon provides fast and bias-aware quantification of transcript expression. Nat. Methods *14*, 417–419.

Perez-Shibayama, C., Gil-Cruz, C., Cheng, H.W., Onder, L., Printz, A., Mörbe, U., Novkovic, M., Li, C., Lopez-Macias, C., Buechler, M.B., et al. (2018). Fibroblastic reticular cells initiate immune responses in visceral adipose tissues and secure peritoneal immunity. Sci. Immunol. *3*, https://doi.org/10.1126/sciimmunol.aar4539.

Platell, C., Cooper, D., Papadimitriou, J.M., and Hall, J.C. (2000). The omentum. World J. Gastroenterol. *6*, 169–176.

Preibisch, S., Saalfeld, S., and Tomancak, P. (2009). Globally optimal stitching of tiled 3D microscopic image acquisitions. Bioinformatics *25*, 1463–1465.

Rangel-Moreno, J., Moyron-Quiroz, J.E., Carragher, D.M., Kusser, K., Hartson, L., Moquin, A., and Randall, T.D. (2009). Omental milky spots develop in the absence of lymphoid tissue-inducer cells and support B and T cell responses to peritoneal antigens. Immunity *30*, 731–743.

Rodda, L.B., Lu, E., Bennett, M.L., Sokol, C.L., Wang, X., Luther, S.A., Barres, B.A., Luster, A.D., Ye, C.J., and Cyster, J.G. (2018). Single-Cell RNA Sequencing of Lymph Node Stromal Cells Reveals Niche-Associated Heterogeneity. Immunity *48*, 1014–1028.e6.

Schauer, C., Janko, C., Munoz, L.E., Zhao, Y., Kienhöfer, D., Frey, B., Lell, M., Manger, B., Rech, J., Naschberger, E., et al. (2014). Aggregated neutrophil extracellular traps limit inflammation by degrading cytokines and chemokines. Nat. Med. *20*, 511–517.

Spallanzani, R.G., Zemmour, D., Xiao, T., Jayewickreme, T., Li, C., Bryce, P.J., Benoist, C., and Mathis, D. (2019). Distinct immunocyte-promoting and adipocyte-generating stromal components coordinate adipose tissue immune and metabolic tenors. Sci. Immunol. *4*, https://doi.org/10.1126/sciimmunol.aaw3658.

Street, K., Risso, D., Fletcher, R.B., Das, D., Ngai, J., Yosef, N., Purdom, E., and Dudoit, S. (2018). Slingshot: cell lineage and pseudotime inference for single-cell transcriptomics. BMC Genomics *19*, 477.

Stuart, T., Butler, A., Hoffman, P., Hafemeister, C., Papalexi, E., Mauck, W.M., 3rd, Hao, Y., Stoeckius, M., Smibert, P., and Satija, R. (2019). Comprehensive Integration of Single-Cell Data. Cell *177*, 1888–1902.e21.

Zhang, N., Czepielewski, R.S., Jarjour, N.N., Erlich, E.C., Esaulova, E., Saunders, B.T., Grover, S.P., Cleuren, A.C., Broze, G.J., Edelson, B.T., et al. (2019). Expression of factor V by resident macrophages boosts host defense in the peritoneal cavity. J. Exp. Med. *216*, 1291–1300.

Zhu, A., Ibrahim, J.G., and Love, M.I. (2019). Heavy-tailed prior distributions for sequence count data: removing the noise and preserving large differences. Bioinformatics *35*, 2084–2092.

STAR★METHODS

KEY RESOURCES TABLE

REAGENT or RESOURCE	SOURCE	IDENTIFIER
Antibodies		
Rat IgG2a anti-mouse CXCL1 antibody, clone 48415	Invitrogen	Cat# MA5-23745 RRID:AB_2609463
Rat IgG2a isotype control antibody	Invitrogen	Cat# 02-9688 RRID:AB_2532970
Armenian hamster anti-mouse TCR beta chain antibody, clone h57-597	Biolegend	Cat# 109229 RRID:AB_10933263
Donkey anti-goat IgG, polyclonal antibody	Invitrogen	Cat# A-11055 RRID:AB_2534102
Donkey anti-rabbit IgG, polyclonal antibody	Invitrogen	Cat# A-31572 RRID: AB_162543
Donkey anti-Mouse IgM-Rhodamine Red-X-AffiniPure F(ab')2 Fragment, μ Chain Specific polyclonal antibody	Jackson ImmunoResearch	Cat# 715-296-020 RRID:AB_2340833
Goat anti-mouse Cathepsin C polyclonal antibody	R&D Systems	Cat# AF1034 RRID:AB_2245504
Goat anti-mouse CCL11/Eotaxin polyclonal antibody	R&D Systems	Cat# AF420 RRID:AB_354486
Goat anti-human ENPP2/Autotaxin polyclonal antibody	R&D Systems	Cat# AF5255 RRID:AB_2277989
Goat anti-human/mouse Myeloperoxidase/MPO polyclonal antibody	R&D Systems	Cat# AF3667 RRID:AB_2250866
Mouse anti-human CD3, clone HIT3a	Biolegend	Cat# 300308 RRID:AB_314044
Mouse anti-human CD3, clone OKT3	Biolegend	Cat# 317342 RRID:AB_2563410
Mouse anti-human CD14, clone HCD14	Biolegend	Cat# 325616 RRID:AB_830689
Mouse anti-human CD19, clone HIB19	Biolegend	Cat# 302208 RRID:AB_314238
Mouse anti-human CD19, clone HIB19	Biolegend	Cat# 302216 RRID:AB_314246
Mouse anti-human CD45, clone HI30	Biolegend	Cat# 304044 RRID:AB_2563812
Mouse anti-human CD16, clone 3G8	Biolegend	Cat# 302015 RRID:AB_314215
Mouse anti-human CD56, clone MEM-188	Biolegend	Cat# 304605 RRID:AB_314447
Mouse anti-human CD15, clone W6D3	Biolegend	Cat# 323038 RRID:AB_2564103
Rabbit anti-mouse Gro alpha/CXCL1 antibody polyclonal	Abcam	Cat# ab86436 RRID:AB_2087574
Rabbit anti-histone H3 (Citrulline 2 + 8+ 17) polyclonal antibody	Abcam	Cat# ab510 RRID:AB_304752
Rabbit anti-ISG15 polyclonal antibody	Invitrogen	Cat# PA5-17461 RRID AB_10979338
Rat anti-mouse MHC Class II (I-A/I-E) antibody, clone M5/114.15.2	Invitrogen	Cat# 47-5321-82 RRID: AB_1548783
Rat anti-mouse/human CD11b antibody, clone M1/70	Biolegend	Cat# 101256 RRID:AB_2563648

(*Continued on next page*)

Continued

REAGENT or RESOURCE	SOURCE	IDENTIFIER
Rat anti-mouse/human CD11b antibody, clone M1/70	Biolegend	Cat# 101222<br>RRID:AB_493705
Rat anti-mouse CD19 antibody, clone 6D5	Biolegend	Cat# 115549<br>RRID:AB_2751271
Rat anti-mouse CD31 antibody, clone 390	Biolegend	Cat# 102421<br>RRID:AB_10613457
Rat anti-mouse CD41 antibody, clone MWReg30	Biolegend	Cat# 133927<br>RRID:AB_2572131
Rat anti-mouse CD44 antibody, clone IM7.8.1	Miltenyi	Cat# 130-102-606<br>RRID:AB_2658181
Rat anti-mouse CD45 antibody, clone 104	Biolegend	Cat# 109836<br>RRID:AB_2563065
Rat anti-mouse CD45 antibody, clone 104	Biolegend	Cat# 109824<br>RRID:AB_830789
Rat anti-mouse CD55 antibody clone REA300	Miltenyi	Cat# 130-104-026<br>RRID:AB_2658705
Rat anti-mouse CD200 antibody, clone OX90	Biolegend	Cat# 123807<br>RRID:AB_2275651
Rat anti-CXCL13 antibody, clone DS8CX13	Thermofisher	Cat# 17-7981-82<br>RRID:AB_2762702
Rat anti-F4/80 antibody, clone BM8	eBiosciences	Cat# 25-4801-82<br>RRID:AB_469653
Rat anti-Ly6C antibody, clone HK1.4	Biolegend	Cat# 128024<br>RRID:AB_10643270
Rat anti-Ly6G antibody, clone 1A8	Biolegend	Cat# 127610<br>RRID:AB_1134159
Rat anti-Ly6G antibody, clone 1A8	Biolegend	Cat# 127627<br>RRID:AB_10897944
Rat anti-Ly6C/Ly6G purified antibody, clone RB6-8C5	Biolegend	Cat# 108453<br>*RRID*:AB_2616681
Rat anti-mouse CD140a antibody, clone APA5	Biolegend	Cat# 135905<br>RRID:AB_1953268
Rat anti-mouse Siglec-F antibody, clone E50-2440	BD Biosciences	Cat# 562681<br>RRID:AB_2722581
Rat anti-mouse Ter119 antibody, clone TER-119	Biolegend	Cat# 116223<br>RRID:AB_2137788
Syrian hamster anti-mouse podoplanin, clone 8.1.1	Biolegend	Cat# 127406<br>RRID:AB_2161930
Syrian hamster anti-mouse podoplanin, clone 8.1.1	Biolegend	Cat# 127410<br>*RRID*:AB_10613649
Syrian hamster anti-mouse podoplanin, clone 8.1.1	Biolegend	Cat# 127412<br>*RRID*:AB_10613648
Bacterial and Virus Strains		
Escherichia coli K12 bioparticles	Invitrogen	E13231
mCherry *Escherichia coli*	Amarh et al., 2018	N/A
Zymosan-A *S. cerevisiae* bioparticles fluorescein conjugate	Invitrogen	Z8241
Zymosan-A *S. cerevisiae*	Sigma	Z4250
Biological Samples		
Human blood, omentum & peritoneal washings	Royal Infirmary of Edinburgh, NHS Lothian	2016/0035
Chemicals, Peptides, and Recombinant Proteins		
4′,6-diamidino-2-phenylindole	Sigma	D9542

(*Continued on next page*)

Continued

REAGENT or RESOURCE	SOURCE	IDENTIFIER
Collagenase I	Worthington	CLS-1
Collagenase D	Roche	11088858001
GSK484	Cayman chemicals	17488
Critical Commercial Assays		
Chromium Single Cell 3′ library and gel bead kit (v2)	10X Genomics	120267
High capacity cDNA Reverse Transcription Kit	Applied Biosystems	4368814
LEGENDplex Human Inflammation Panel	Biolegend	740879
LEGENDplex Human Pro-inflammatory Chemokine	Biolegend	740003
LEGENDplex Mouse Pro-inflammatory chemokine panel	Biolegend	740451
LIVE/DEAD Fixable Blue Dead Cell Stain Kit	Invitrogen	L23105
NovaSeq 6000 S1 Reagent Kit	Illumina	20012864
RNeasy plus micro Kit	QIAGEN	74034
ToxinSensor^{TM} Chromogenic LAL Endotoxin Assay Kit	Genscript	L00350
Triton X-100	Sigma	X100
SYTOX Blue nucleic acid stain	Invitrogen	S11384
Deposited Data		
scRNaseq dataset	GEO	GSM4053741
Experimental Models: Organisms/Strains		
Mouse: C57BL/6JOlaHsd	Bred in house at University of Edinburgh animal facilities	N/A
Mouse: *Elane*−/− (C57BL/6J)	Belaaouaj et al., 1998	N/A
Oligonucleotides		
Cxcl1 Taqman gene expression assay	Thermofisher	Mm04207460_m1
Gapdh Taqman gene expression assay	Thermofisher	Mm9999995_g1
Software and Algorithms		
FlowJo 10	FLOWJO, LLC	https://www.flowjo.com
Prism 7	GraphPad Software	https://www.graphpad.com/scientific-software/prism
Fiji	ImageJ	https://imagej.net/Fiji
R	The R Foundation	www.r-project.org
Scmap version 1.8.0	Kiselev et al., 2018	https://bioconductor.org/packages/release/bioc/html/scmap.html
Salmon version 0.14.1	Patro et al., 2017	https://github.com/COMBINE-lab/salmon/releases
DESeq2	Love et al., 2014	https://bioconductor.org/packages/release/bioc/html/DESeq2.html
apeglm	Zhu et al., 2019	https://bioconductor.org/packages/release/bioc/html/apeglm.html
EnhancedVolcano version 1.4.0	Blighe and Lewis, 2019	http://bioconductor.org/packages/release/bioc/html/EnhancedVolcano.html
slingshot	Street et al., 2018	https://github.com/kstreet13/slingshot
Seurat R package version 3.1.1	Stuart et al., 2019	https://github.com/satijalab/seurat/releases/tag/v3.1.1
Cell Ranger v3.0.2 Single-Cell Software Suite	10X Genomics	https://support.10xgenomics.com/single-cell-gene-expression/software/pipelines/latest/installation
Bioreactome	Fabregat et al., 2018	https://reactome.org
Huygens 19.0 software	Scientific Volume Imaging	https://svi.nl/Huygens-Software
LAS-X-3D	Leica	https://www.leica-microsystems.com/products/microscope-software/p/leica-las-x-ls/downloads/

RESOURCE AVAILABILITY

Lead Contact

Further information and requests for resources and reagents should be directed to and will be fulfilled by the Lead Contact, Cecile Benezech (cbenezec@ed.ac.uk)

Materials availability

This study did not generate new unique reagents

Data and Code Availability

The accession number for the scRNaseq dataset reported in this paper is GEO: GSM4053741. The authors declare that all relevant data supporting the findings of this study are available on request. R scripts for performing the main steps of analysis are available from the Lead contact on reasonable request.

EXPERIMENTAL MODEL AND SUBJECT DETAILS

Experimental animals

All experiments were conducted under a license granted by the Home Office (UK) that was approved by the University of Edinburgh or Lancaster University animal welfare and ethics review board. All individual experimental protocols were approved by a named veterinarian surgeon prior to the start of the experiment. Experiments were performed using female C57BL/6 (C57BL/6JOlaHsd) or NE deficient *Elane*$^{-/-}$ mice (Belaaouaj et al., 1998) aged 8-12 weeks. All animals were bred and maintained under specific pathogen–free conditions at the University of Edinburgh or Lancaster University Animal Facilities.

Human subjects

This study was approved by the Regional Research Ethics Committee (SE. Scotland REC 02; 16/SS/0042), the University of Edinburgh/NHS Lothian ACCORD R and D Office (ref: 2016/0035) and the Office of the Caldicott Guardian, NHS Lothian (patient confidentiality advocate). Individuals were recruited after informed, signed consent was obtained. Clinical data (Table S1) and samples were collected from patients undergoing laparoscopic surgery under general anesthesia for the following indications: biliary colic and possible or suspected appendicitis, at the Royal Infirmary of Edinburgh between 1st April 2016 and 30th June 2018. After the induction of anesthesia, a single tube of blood was collected into a BD Vacutainer prefilled by the manufacturer with the anticoagulant EDTA. At operation, and as soon as practical and safe after the insertion of the laparoscopic ports (to avoid iatrogenic contamination with blood), 25mL of sterile 0.154 M NaCl solution was washed into the area of interest and then aspirated using a sputum trap interposed into the surgical suction equipment. Next a 5 cm^3 sample of omentum was resected with scissors to avoid diathermy artifact, retrieved and the sample site haemostasis ensured with diathermy. A 2 cm^2 sample of parietal peritoneum adjacent to a port site was obtained and haemostasis ensured. Operations then continued as planned. Clinical samples were handled as follows: omentum and parietal peritoneum samples were collected into 20 mL of sterile dPBS (Sigma) within a 50ml tube. Peritoneal washings were placed into a sterile 50ml tube. All patient samples were stored on wet ice or at 4°C prior to collection from the clinical research facility and transported to the research laboratory on foot, samples collected after 5pm were processed the following day. If there were any unexpected findings at surgery, e.g., free peritoneal blood or peritoneal malignancy, patients were removed from the study and no tissue samples were taken for research purposes. There were no adverse effects due to the research study conduct.

METHOD DETAILS

Peritonitis models

To induce peritonitis, mice were injected intra-peritoneally with either 0.5mg Zymosan-A (Sigma) in PBS, 0.25mg of Fluorescein labeled Zymosan-A (Invitrogen), or PBS alone and samples were isolated 2-72 h later. To block NETosis, mice were injected i.p. with 400 μg/mouse of GSK484 (Cayman Chemical). Blocking antibodies against CXCL1 (Clone 48415, Invitrogen) or isotype control Rat IgG2a (Invitrogen) antibodies were injected i.p. 2 h after induction of peritonitis (40 μg/mouse). Following Zymosan and anti-CXCL1 treatment, omenta were isolated and cultured with mCherry *E. coli* (Amarh et al., 2018) or *E. coli* bioparticles (Invitrogen) *in vitro* for 5 min prior to thorough washing with RPMI-1640 (Sigma), and wholemount staining as described below.

Peritoneal exudate cells (PEC) were isolated by flushing murine peritoneal cavities with RPMI 1640 (Sigma). Murine omenta were enzymatically digested with 1mg/mL Collagenase D (Roche) for 35 min at 37°C in RPMI 1640 (Sigma) containing 1% Fetal Bovine Serum (FBS) (Sigma). Spleens were mechanically disrupted using glass slides.

Human sample preparation

Human Omentum was weighed and 0.035 – 1.8g of tissue was digested using 2mg/mL Collagenase I (Worthington) in PBS (Invitrogen/sigma) 2% Bovine Serum Albumin (BSA, Sigma), samples were disrupted using an Octolyser (Miltenyi), incubated at 37°C with intermittent shaking for 45 min, subjected to a second Octolyser dissociation step, ions were chelated by addition of EDTA (0.5M, Sigma), samples were filtered through a 100 μM filter (BD) and washed with 20ml of 2%BSA PBS prior to centrifugation at

1700pm for 10 min. The cell pellet was resuspended in 2ml of PBS 2% BSA for flow-cytometric analysis. Peritoneal wash and blood samples were centrifuged, the supernatant and serum were collected for further analysis and the cell pellet was resuspended for flow-cytometric analysis. Cell numbers and live cell count were determined using a BioRad TC20 automated cell counter and 0.4% Trypan Blue (Sigma). For the omentum *ex vivo* culture, a small piece of omentum (between 0.02 and 0.06 g) was placed in culture in 1ml of RPMI 1640 (Sigma) 10% fetal bovine serum (Sigma) 2 mM L-glutamine (Sigma) for 2 h at 37°C.

Flow cytometry

Murine cells were stained with LIVE/DEAD (Invitrogen), blocked with mouse serum and anti-murine CD16/32 (clone 2.4G2, Biolegend) and stained for cell surface markers. Human samples were blocked with serum, stained for cell surface markers (See Key Resources Table for list of antibodies used), and DAPI (Sigma) was added to the cells prior to acquisition. All samples were acquired using a BD Fortessa and analyzed with FlowJo software (FlowJo, LLC).

Cell-sorting and quantitative real-time PCR

Cells were stained for cell surface marker and sorted using a FACS Aria Fusion directly in 350 μL RLP buffer before RNA extraction using RNeasy Plus Micro Kit (QIAGEN) according to manufacturer's instruction. Complementary DNA for mRNA analysis was synthesized from total RNA using High Capacity cDNA Reverse Transcription Kit (Applied biosystems). *Cxcl1* expression was assessed using TaqManGene Expression Assay (Mm04207460_m1) by qRT-PCR (Life Technologies) and normalized to glyceraldehyde-3-phosphate dehydrogenase (*Gapdh*, Mm9999995_g1). The C_t of *Gapdh* was subtracted from the C_t of *Cxcl1*, and the relative amount was calculated as $2^{-\Delta C}{}_t$. Means of triplicate reactions were represented for n = 4 biological samples per condition from two separate sorts.

Detection of chemokines, cytokine, dDNA and LPS

dsDNA was detected in lavage fluid and omentum culture supernatants using the picogreen assay following the manufacturer's instructions (Invitrogen). LPS was detected in human serum, omentum culture supernatants and peritoneal lavage fluid using the ToxinSensor™ Chromogenic LAL Endotoxin Assay Kit (GenScript) following manufacturer's instructions. Human Pro-inflammatory chemokines and Human Inflammation Legendplex arrays (Biolegend) were used to detect cytokines and chemokines following the manufacturer's instructions. For the heatmap representation in Figure 7C, original values were scaled between 0 and the maximum value detected for each cytokine and presented as a fraction of maximum secretion. Murine samples of omentum, mesenteries, parietal wall, diaphragm and liver were placed in culture for 2 h in 300 μL RPMI containing 10% FBS (Sigma) and 1% L-glutamine (Sigma). The quantity of murine CXCL1, CCL2 and CXCL10 present within cell culture supernatants was determined using a mix and match mouse pro-inflammatory chemokine Legendplex array (Biolegend) following the manufacturer's instructions.

Immunofluorescence staining and microscopy

Human and mouse omentum samples were fixed for one h on ice in 10% NBF (Sigma) and then permeabilized in PBS 1% Triton X-100 (Sigma) for 20 min at room temperature prior to staining with primary antibodies for one h at room temperature in PBS 0.5% BSA 0.5% Triton. After washing in PBS, tissues were stained with secondary antibodies for one h at room temperature in PBS 0.5% BSA 0.5% Triton. For extra-cellular DNA staining, human omental tissues were first stained with SYTOX Blue (Invitrogen) 1 in 5000 in RPMI 1640 (Sigma) for 30 min at room temperature, washed in RPMI and then fixed in 10% NBF (Sigma) prior to permeabilization and staining. Antibodies used are listed in Key Resources Table. After mounting with Fluoromount G (Thermofisher), confocal images were acquired using a Leica TCS SP5 or TCS SP8 laser scanning confocal microscope. Image analysis was performed using Fiji and 3D reconstruction was created using LAS-X-3D (Leica). The mean gray value of MPO, Ly6G, CitH3 and mCherry *E. coli* was calculated inside a perimeter delimited manually as the border of the cluster. To calculate FALC volume, we manually assessed the maximum length, width and depth (z) of the clusters visualized with DAPI using 10x objective while scanning on SP5 confocal microscope. To calculate the area of FALCs covered by *E. coli,* the perimeter of the FALC was delimited manually and a fixed threshold for *E.coli* fluorescence was set. The number of CitH3$^+$ nuclei and *E. coli* bioparticles was calculated using the "analyse particles" function of Fiji. The large picture of the omentum was taken as stack on a Zeiss Axio Observer Z1, deconvolved using the SVI Huygens 19.0 software and processed in Fiji first using the Maximum Intensity Projection and then stitched using Grid/Collection stitching (Preibisch et al., 2009).

Droplet based scRNaseq and data pre-processing

Immediately post-sorting, DAPI$^-$CD45$^-$Ter119$^-$CD41$^-$CD31$^-$ stromal cell pooled from the omenta from three naive mice were run on the 10X chromium (10X Genomics) and then through library preparation following the recommended protocol for the Chromium Single Cell 3′ Reagent Kit (v2). Libraries were run on the NovaSeq S1for Illumina sequencing. Sequence reads were processed using the Cell Ranger v3.0.2 Single-Cell Software Suite from 10x Genomics. Reads were aligned to the mm10 mouse references genome (Ensembl 93). As a quality control step, genes were excluded if they were expressed in fewer than three cells. Cells were excluded on a number of criteria: those with fewer than 300 genes (n = 13), those with fewer than 300 or greater than 16000 UMIs (n = 21) or those with mitochondrial gene proportion of over 20% of total UMI counts (n = 14). A global normalization was performed where gene expression was normalized for each cell based on its total expression before being multiplied by a scale factor of 10,000 and log transformed. Variation in the UMI counts of each cell was regressed using a linear regression. Residuals from this model were centered and scaled by subtracting the average expression of each gene followed by dividing by the standard deviation of each

gene. A list of 2000 variable genes were generated using the 'vst' method of the *FindVariableFeatures* function from the *Seurat* R package version 3.1.1 (Stuart et al., 2019). We obtained the transcriptional profile of 4,501 cells that passed quality control and filtering, for which we measured a median of 2,214 genes per cell.

scRNaseq data analysis and visualization

Dimensionality reduction, unsupervised clustering and differential gene expression were performed using *Seurat.* We used between 1 and 10 principal components for shared nearest neighbor (SNN) clustering, as determined by the dataset variability shown in the principle component analysis (PCA). The resolution parameter was optimized based on the number of resulting clusters. Clusters were initially categorised into cell lineages based on expression of known marker genes. Clusters annotated as endothelial (*Pecam1, Cdh5*), immune (*Ptprc*), proliferating (*Mki67, Pcna, Top2a*) or those with a median number of genes per cell below 1000 were excluded from further analysis. The final dataset contained 3,838 cells measuring a median of 2,249 genes per cell. Variable features and scaled expression were re-calculated for the refined dataset, followed by dimensionality reduction and re-clustering using the same methodology described above. Differential gene expression analysis was conducted using the *FindAllMarkers* function of *Seurat* and a Wilcox rank sum test (Table S3). Only genes with at least a 0.25 log-fold change and expressed in at least 25% of the cells in the cluster under comparison were evaluated. The *FindMarkers* function was used for direct cluster-to-cluster comparison using the same statistical model and thresholds. Cluster similarity was assessed using the *BuildClusterTree* function of *Seurat*. All violin plots, UMAP visualizations and heatmaps were generated using functions from *Seurat, ggplot2, pheatmap, and grid* R packages. UMAPs were constructed using the same number of principle components as the corresponding clustering. Heatmaps were generated using scaled expression and their range was clipped from −2.5 to 2.5. DEGs from each cluster were used for pathway analysis using Bioreactome (Fabregat et al., 2018)

Mesothelium trajectory inference

We generated a subset of the data including clusters "*Cxcl13*$^+$ mesothelium," "*Ifit*$^+$ mesothelium," and "Mesothelium," re-scaled the expression data, and performed PCA analysis followed by pseudotime analysis in *slingshot* (Street et al., 2018). Lineage inference was performed using a cluster-based minimum spanning tree on PCs 1:10, specifying "Mesothelium" as the starting cluster. Pseudotime values thus generated were mapped to the previously generated UMAP for visualization. Variable genes of the subsetted data were then regressed on the resulting pseudotime variables using a general additive model to identify those genes that are differentially expressed across pseudotime. Cubic smoothing spline curves were fitted to the scaled expression of a selection of the top 50 differentially expressed genes (p value < 1e-16) along the pseudotemporal trajectory using the *smooth.spline* (df = 3) command from the R *stats* package. These results were plotted as a heatmap with the range clipped from −2 to 2.

Mapping mesenteric single cell data

The processed single cell mesenteric dataset was downloaded from GEO: GSE102665 (Koga et al., 2018). Cell filtering, normalization and dimensionality reduction from the omentum scRNaseq data analysis (as above) were replicated on the mesenteric scRNaseq data. *Pdgfra*$^+$*Pdpn*$^-$ and *Pdgfra*$^+$*Pdpn*$^+$ clusters were identified, isolated and mapped individually to the omentum dataset using *Scmap* version 1.8.0 (Kiselev et al., 2018), following instructions given in the package vignette for feature selection and indexing of the omentum data, and cluster mapping.

RNaseq data analysis

RNaseq data for epididymal adipose mesothelial cells was downloaded from GEO: GSM3754627, GSM3754628, GSM3754629. RNaseq data for omentum mesothelial cells was downloaded from GEO: GSM3754642, GSM3754643, GSM3754644 (Buechler et al., 2019). We used *Salmon* version 0.14.1 (Patro et al., 2017) to align and quantify transcripts to the GRCm38 reference transcriptome (ensemble 81) with "validateMappings," "seqBias," and "gcBias" options enabled. This data was imported into R using the *tximeta* package (citation), and genes with a count level of 1 or less were removed. *DESeq2* (Love et al., 2014) was used to perform differential gene expression analysis between the omentum and epididymal samples with an adjusted p value cutoff set to 0.05 for a log fold change threshold of 0. Log-fold change shrinking was performed using the *apeglm* method (Zhu et al., 2019) before plotting a volcano plot in *EnhancedVolcano* version 1.4.0 (Blighe and Lewis, 2019) where y axis (-$\log_{10}$ adjusted P) were clipped to 50.

QUANTIFICATION AND STATISTICAL ANALYSIS

No randomization and no blinding was used for the animal experiments. Whenever possible, the investigator was partially blinded for assessing the outcome (bacterial binding). All data were analyzed using Prism 7 (GraphPad Prism, La Jolla, CA). Statistical tests performed, sample size and number of repetitions for each dataset, are described within the relevant figure legend.

Immunity, Volume *52*

Supplemental Information

Stromal Cells Covering Omental Fat-Associated Lymphoid Clusters Trigger Formation of Neutrophil Aggregates to Capture Peritoneal Contaminants

Lucy Helen Jackson-Jones, Peter Smith, Jordan Raymond Portman, Marlène Sophie Magalhaes, Katie Jude Mylonas, Matthieu Marie Vermeren, Mark Nixon, Beth Emily Pollot Henderson, Ross Dobie, Sonja Vermeren, Laura Denby, Neil Cowan Henderson, Damian James Mole, and Cécile Bénézech

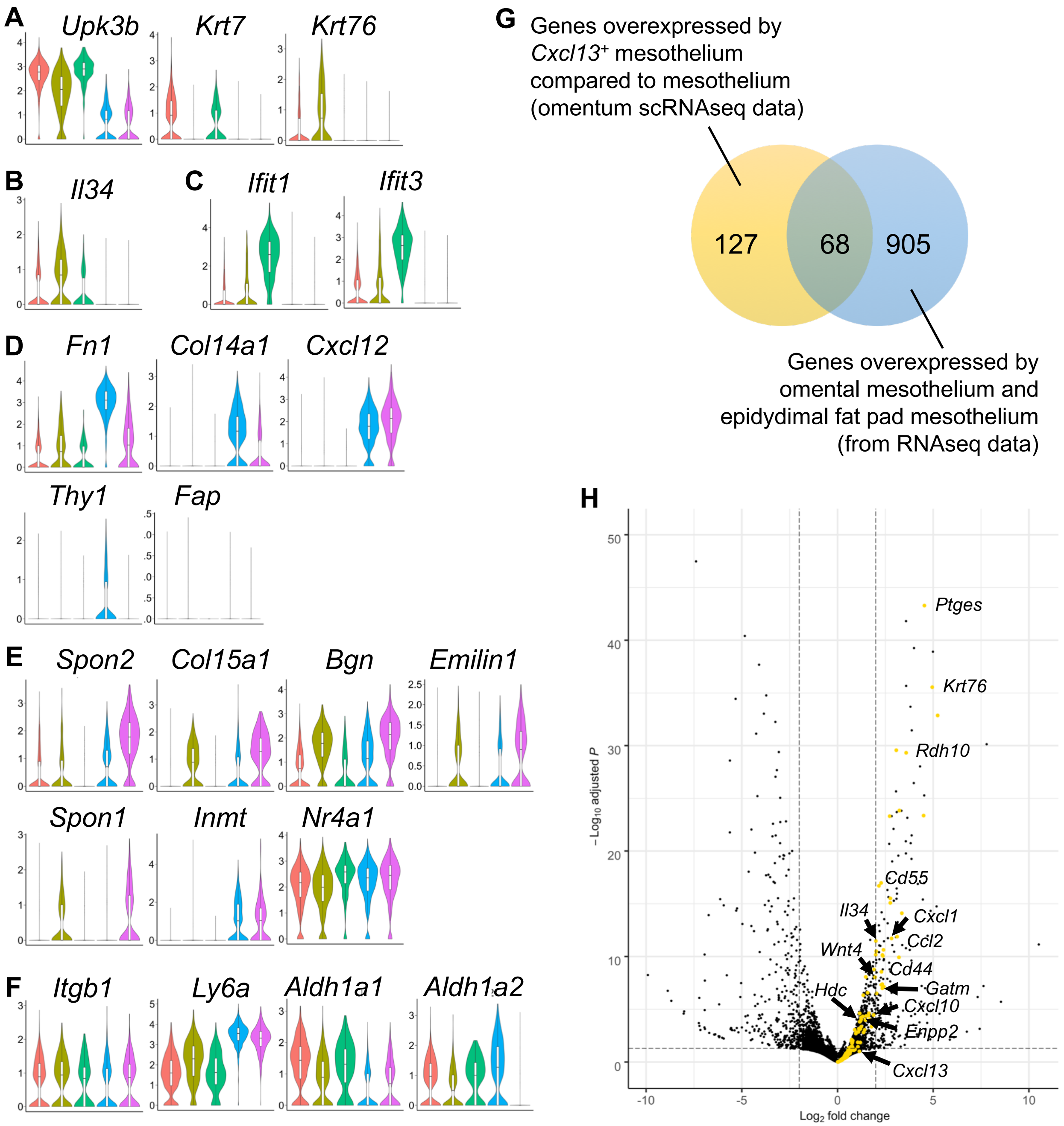

Figure S1. Related to Figure 1. (A-F) Violin plots of canonical omental stromal cell gene expression by cluster with highest log-normalized expression value labelled. Plots highlighting DEGs characteristic of mesothelial (**A, F**) immune (**B, C**) fibroblast (**D**) and ECM (**E**). **(G-H) Differential gene expression analysis between omentum mesothelial cells and epidydimal mesothelial cells. G,** Venn Diagram showing the number of genes significantly upregulated (adjusted p value <0.05) by the omental *Cxcl13*$^{+}$ mesothelial cell cluster compared to the omental mesothelial cell cluster (yellow circle, 195 total) and by *PDPN*$^{+}$*PDFGRa*$^{-}$ omental mesothelial cells compared to *PDPN*$^{+}$*PDFGRa*$^{-}$ epidydimal fat pad mesothelial cells (blue circle, 973 total) obtained from published RNAseq data GSM3754642, GSM3754643, GSM3754644, GSM3754627, GSM3754628, and GSM3754629) (Buechler et al., 2019). 68 DEG overlapping. **H**, Volcano plot showing DEG between omentum mesothelial cells and epididymal mesothelial cells in grey. Examples of genes that are also upregulated by the omental *Cxcl13*$^{+}$ mesothelial cell cluster compared to the omental mesothelial cell cluster are shown in yellow.

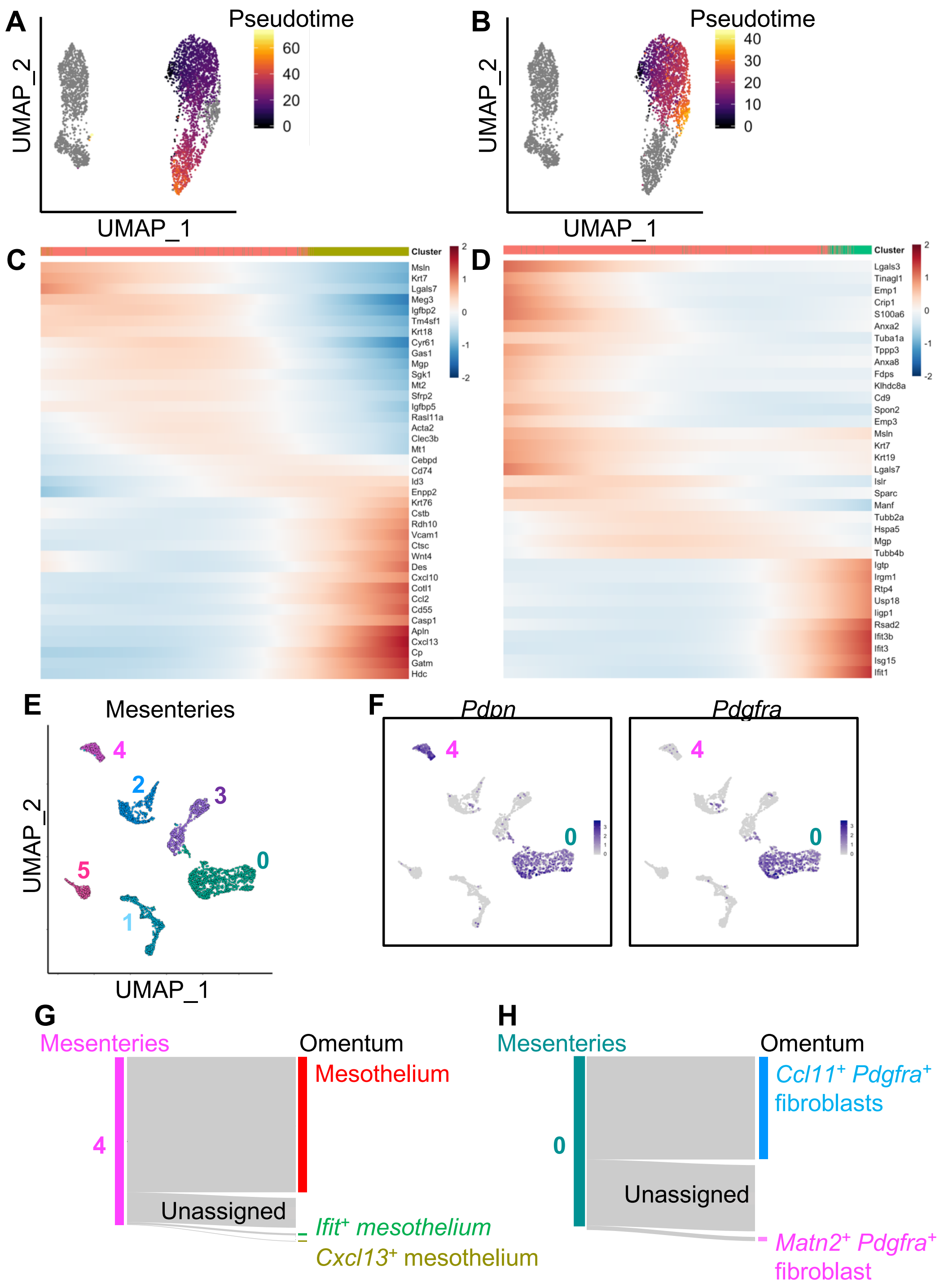

Figure S2. Mesothelium trajectory inference and mapping of mesenteric stromal cell subsets to omental stromal cell subsets related to Figure 1. A-B, UMAP visualization on (top panel) of the Pseudotime values from Mesothelium (starting point) to *Cxcl13*$^{+}$ mesothelium (**A**) and *Ifit*$^{+}$ mesothelium (**B**). **C-D**, Heat map with spline curves fitted to genes differentially expressed along a trajectory from mesothelium to *Cxcl13*$^{+}$ mesothelium (**C**) and *Ifit*$^{+}$ mesothelium (**D**). **E**, Unsupervised clustering of mesenteric cells visualized with UMAP from the published single cell dataset GSE102665 (Koga et al., 2018). **F,** Gene expression of *Pdpn* and *Pdgfra* distinguishing *Pdpn*$^{+}$*Pdgfra*$^{-}$ mesenteric mesothelial cells (cluster 4, pink) and *Pdpn*$^{+}$*Pdgfra*$^{+}$ mesenteric fibroblasts (cluster 0, dark green) projected onto UMAP plots. Color scaled for each gene with highest log-normalized expression level noted. **G-H**, Mapping of the *Pdgfra*$^{+}$*Pdpn*$^{-}$ mesenteric mesothelial cell cluster (**G**) and the *Pdgfra*$^{+}$*Pdpn*$^{+}$ mesenteric fibroblast cluster (**H**) to the omentum scRNAseq dataset.

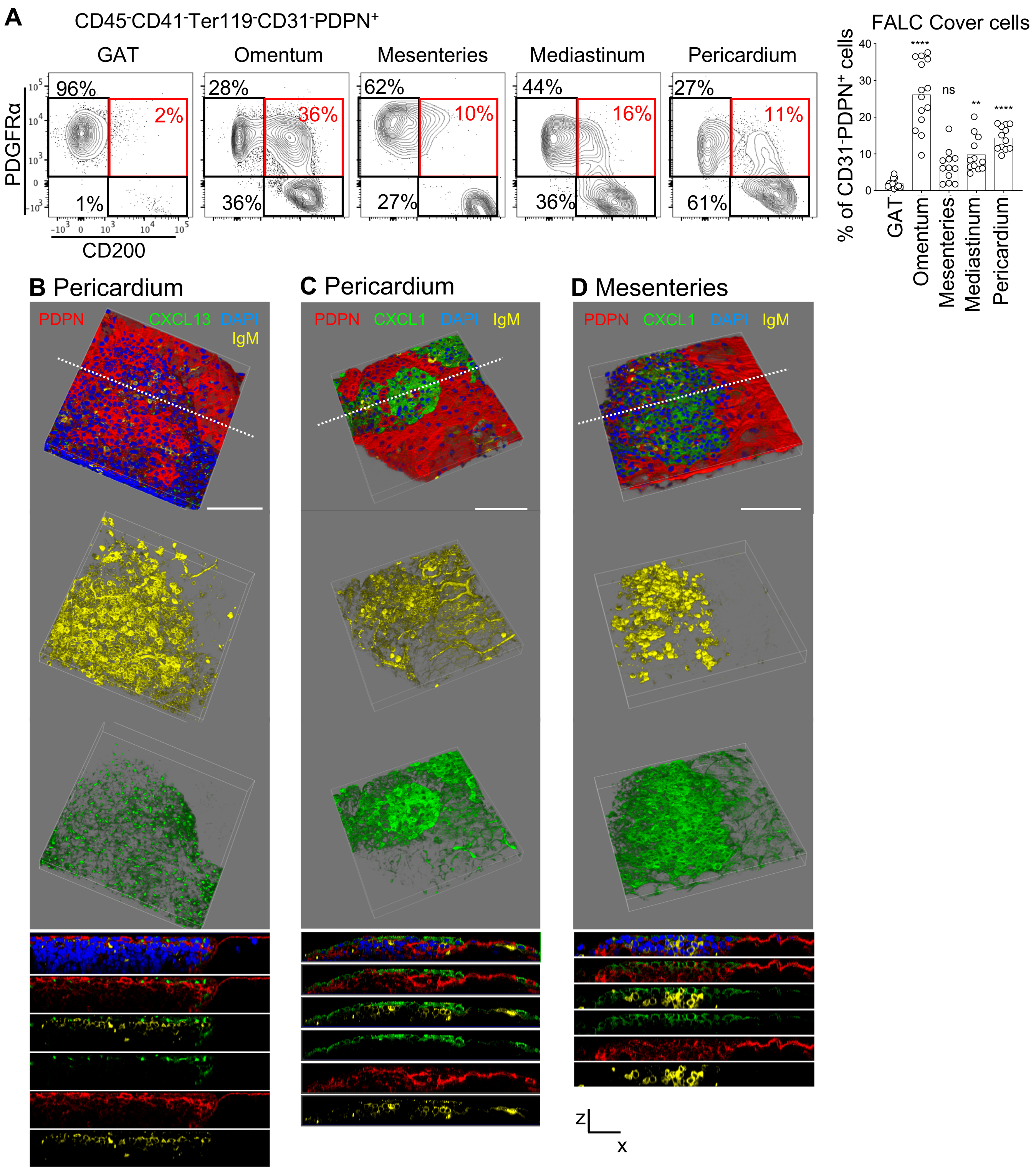

Figure S3. FALC cover cells in other FALC rich visceral adipose tissues related to Figure 1 and 2. A, Representative gating strategy used to determine the percentage of PDPN^{+}PDFGRa^{int}CD200^{int} *Cxcl13*$^{+}$ FALC cover cells (red gate) present in the CD45^{-}CD41^{-}Ter119^{-}CD31^{-}PDPN^{+} non-endothelial stromal cell fraction of the gonadal adipose tissue (GAT), omentum, mesenteries, mediastinum and pericardium. Data pooled from four independent experiments with n=10 mice per group. ANOVA with Sidak's multiple comparisons test were applied after assessing normality using D'Agostino & Pearson Normality test, ns= non-significant, ** P=<0.01, **** P=<0.0001. **B-D,** Representative confocal imaging and 3D reconstruction of a FALC from the pericardium (**B, C**) or the mesenteries (**D**) showing a view of the surface of the cluster and a z section (along the dotted line) of the cluster with PDPN (red), CXCL13 (**B**, green) or CXCL1 (**C**, **D**, green), DAPI (blue) and IgM (red). All staining representative of n≥8 clusters from n≥4 mice in at least 2 independent experiments. Scale bar 100 μm.

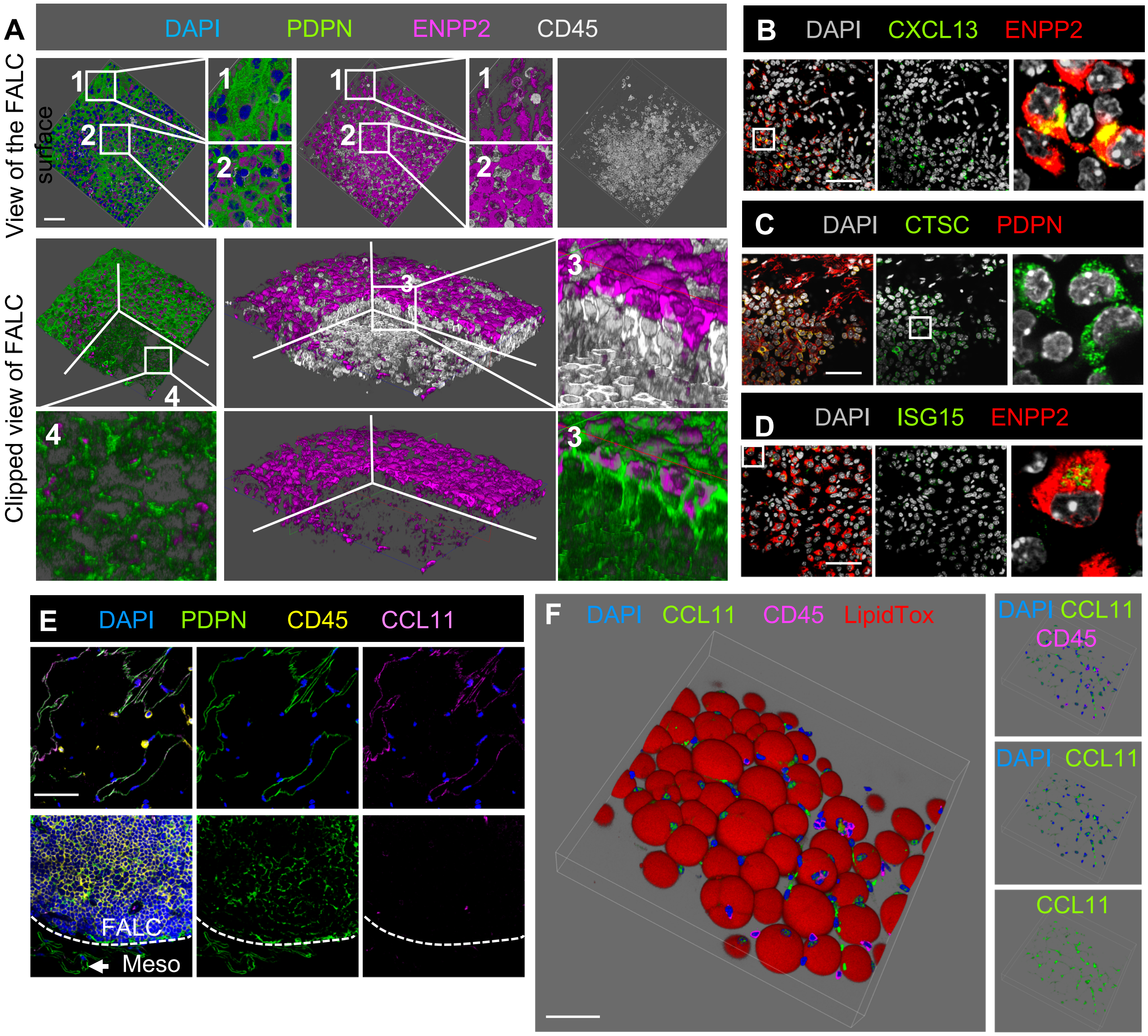

Figure S4. Characterization of FALC stromal cells related to Figure 2. A, 3D reconstruction of omFALC obtained by confocal imaging of wholemount immunofluorescence staining of the omentum showing the surface of the cluster (first row) and two clipped views of the inside of the cluster (second row). Four enlargements are shown: 1 for mesothelial cells, 2 and 3 for FALC cover cells and 4 for FALC FRCs. **B-D,** Representative confocal images of wholemount immuno-fluorescence staining of omentum showing the surface of omFALCs and the expression of CXCL13 (**B**, green), Cathepsin-C (**C**, green) and ISG15 (**D**, green) with DAPI (white), ENPP2 (**A,D** red) and PDPN (**C**, red) by FALC cover cells. Scale Bar 50μM. Staining representative of n≥8 clusters from n≥4 mice in 2 independent experiments. **E,** Representative confocal image of naive whole mount immunofluorescence staining of omentum, DAPI (blue), PDPN (green), CD45 (yellow), CCL11 (magenta). Upper panel showing omental adipose in the absence of FALC, lower panel $CD45^{+}$ FALC within omental adipose. FALC boundary delineated by dotted line in lower panel. Scale bar 50μM. **F**, Representative confocal imaging and 3D reconstruction of adipocyte in omentum with neutral lipid stained with LipidTox (red), CCL11 (green), DAPI (blue) and CD45 (magenta). Scale bar 100 μm. Staining representative of n=8 clusters or field of view from n=4 mice in 2 independent experiments.

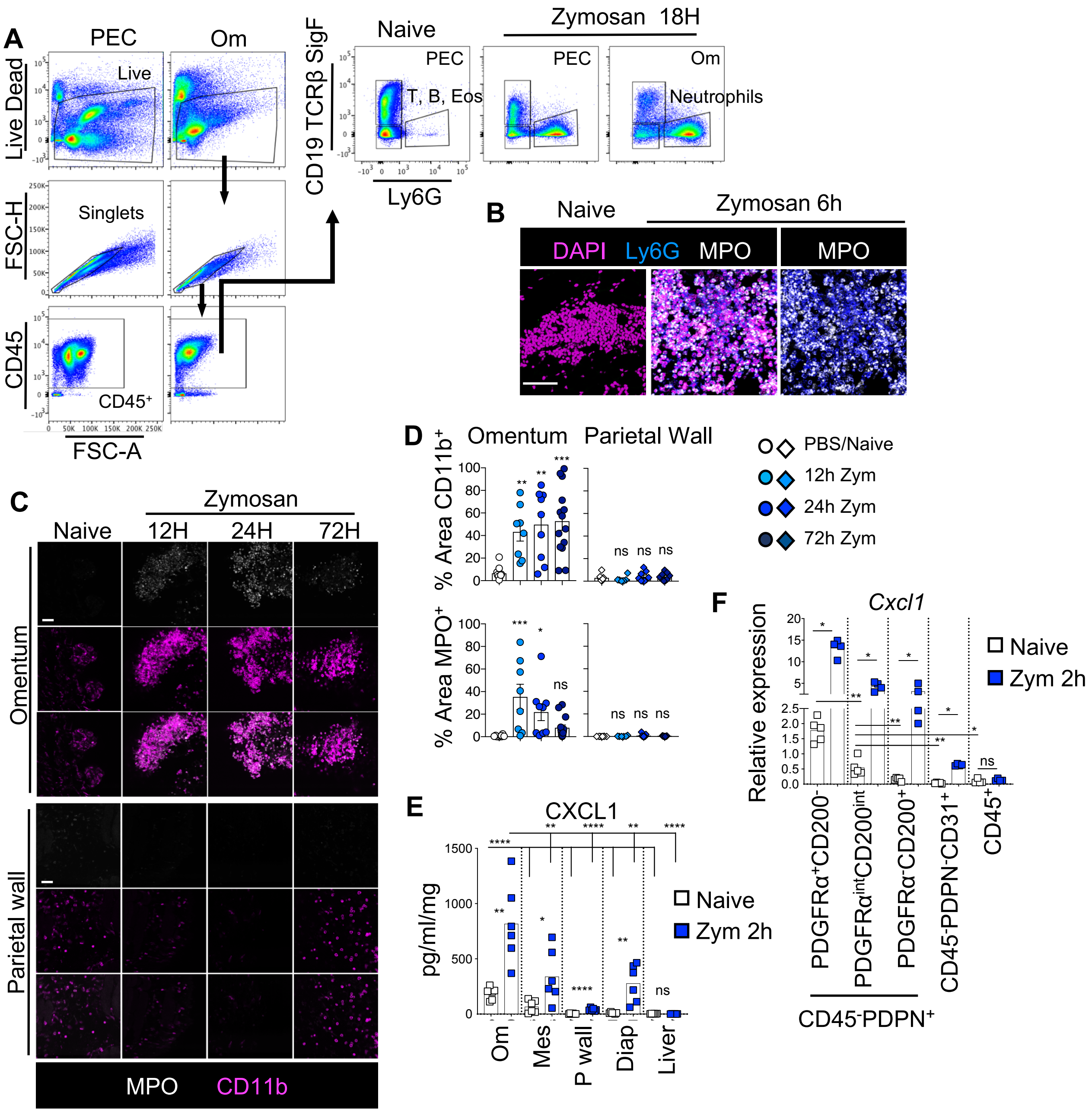

Figure S5. Analysis of neutrophil recruitment in the peritoneal cavity following Zymosan-A injection related to Figure 3. A, Flow-cytometric gating strategy of murine PEC and omentum digests. PEC and omentum were analysed by dead cell exclusion, determination of single cell populations (including B cells) using scatter profiles and based on CD45 positivity. $CD19^+$ B cells, $TCR\beta^+$ T cells and Siglec F^+ eosinophils were excluded, $Ly6G^+$ neutrophils were gated. **B**, Representative confocal images of wholemount immuno-fluorescence staining of omentum from naïve mice and 6 hours post-i.p. injection of Zymosan-A, DAPI (magenta), MPO (White) and Ly6G (Blue). **C-D**, Representative confocal images of wholemount immuno-fluorescence staining of the omentum and the peritoneal surface of the parietal wall from naïve mice and at the indicated time points following i.p. injection of Zymosan with MPO (white) and CD11b (magenta) (**C**) and quantification of the percentage $CD11b^+$ or MPO^+ area (**D**). **E**, Amounts of CXCL1 secreted into the supernatant of 2h omentum, mesenteric, parietal wall, diaphragm or liver explant culture per mg of tissue and per ml from naïve mice (white squares) and 2h after i.p. injection of Zymosan-A (blue squares). Data pooled from two independent experiments with n=6 mice per group. **F**, Relative amounts of *Cxcl1* expressed by the following stromal cell populations ($CD45^-CD41^-Ter119^-$) $PDPN^+CD31^-PDGFRa^+CD200^-$, $PDPN^+CD31^-PDGFRa^{int}CD200^{int}$, $PDPN^+CD31^-PDGFRa^-CD200^+$, $CD31^+$, and $CD45^+$ hematopoietic cells isolated from naïve mice and 2h post-Zymosan A i.p. injection. Data pooled from 2 independent experiments with n=4 biological replicates per group. ANOVA with Sidak's multiple comparisons test (**D**, **E**) or Mann Whitney test (**F**) were applied after assessing normality using Shapiro-Wilk Normality test, ns= non-significant, **** P=<0.0001. All staining representative of n≥8 clusters or areas from n≥4 mice in at least 2 independent experiments. Scale bar 50 μm.

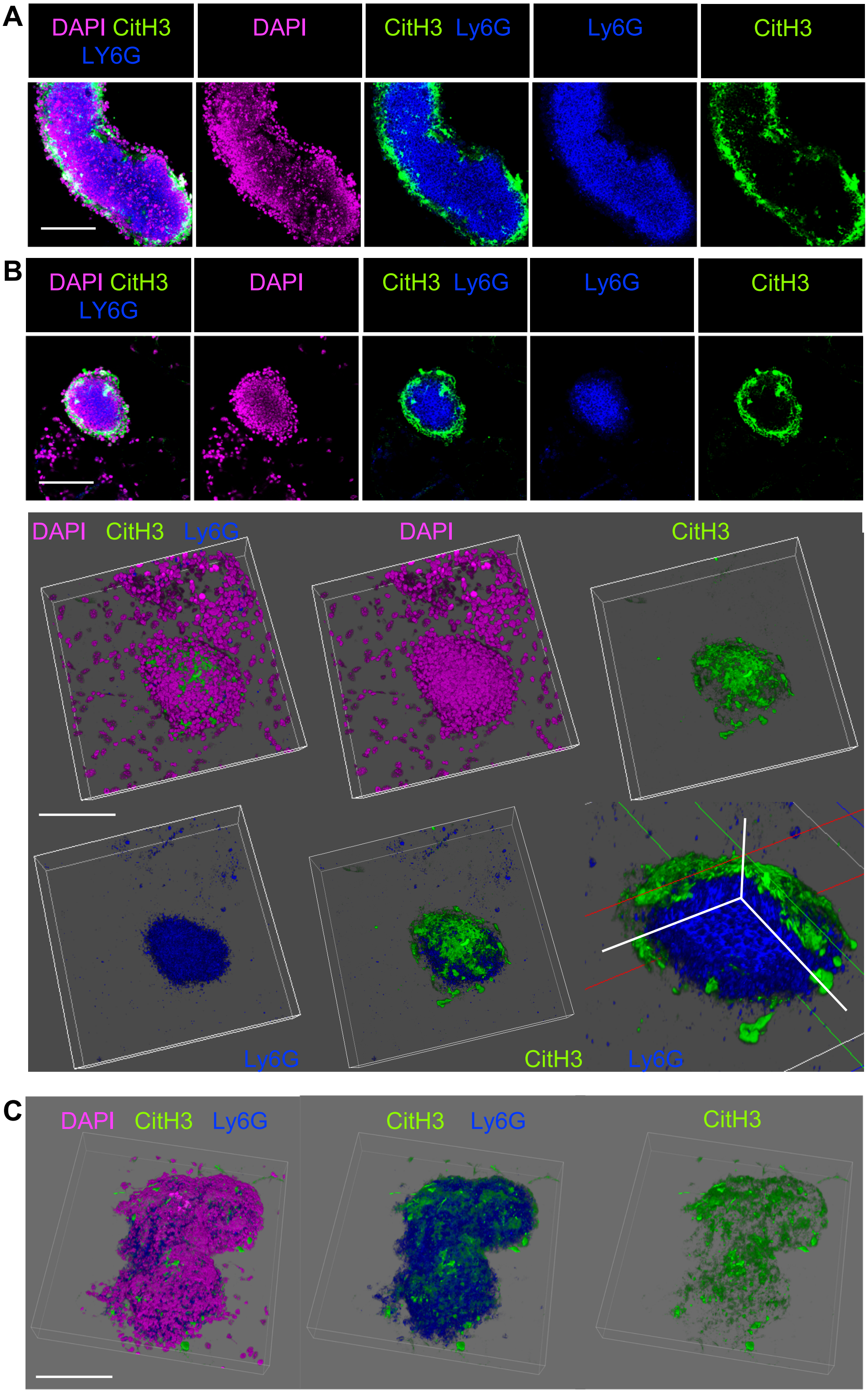

Figure S6. CitH3$^+$ DNA covers neutrophil aggregates forming on omental and mesenteric FALCs during peritonitis related to Figure 4. A, Confocal image showing inside the cluster presented in Fig. 4C obtained by wholemount immuno-fluorescence staining of omentum from mice 6 hours post-Zymosan injection showing DAPI (magenta), CitH3 (Green) and Ly6G (Blue). **B**, Confocal image showing inside another representative cluster 6 hours post-Zymosan injection (first panel) and 3D reconstruction of whole omFALC showing the surface of the cluster (first row) and a clipped view of the inside of the cluster (second row). **C**, Representative 3D reconstruction obtained by wholemount immuno-fluorescence staining of mesenteries from mice 6 hours post-Zymosan injection as in **A**. All staining representative of n≥8 clusters or areas from n≥4 mice in at least 2 independent experiments. Scale bar 100µM.

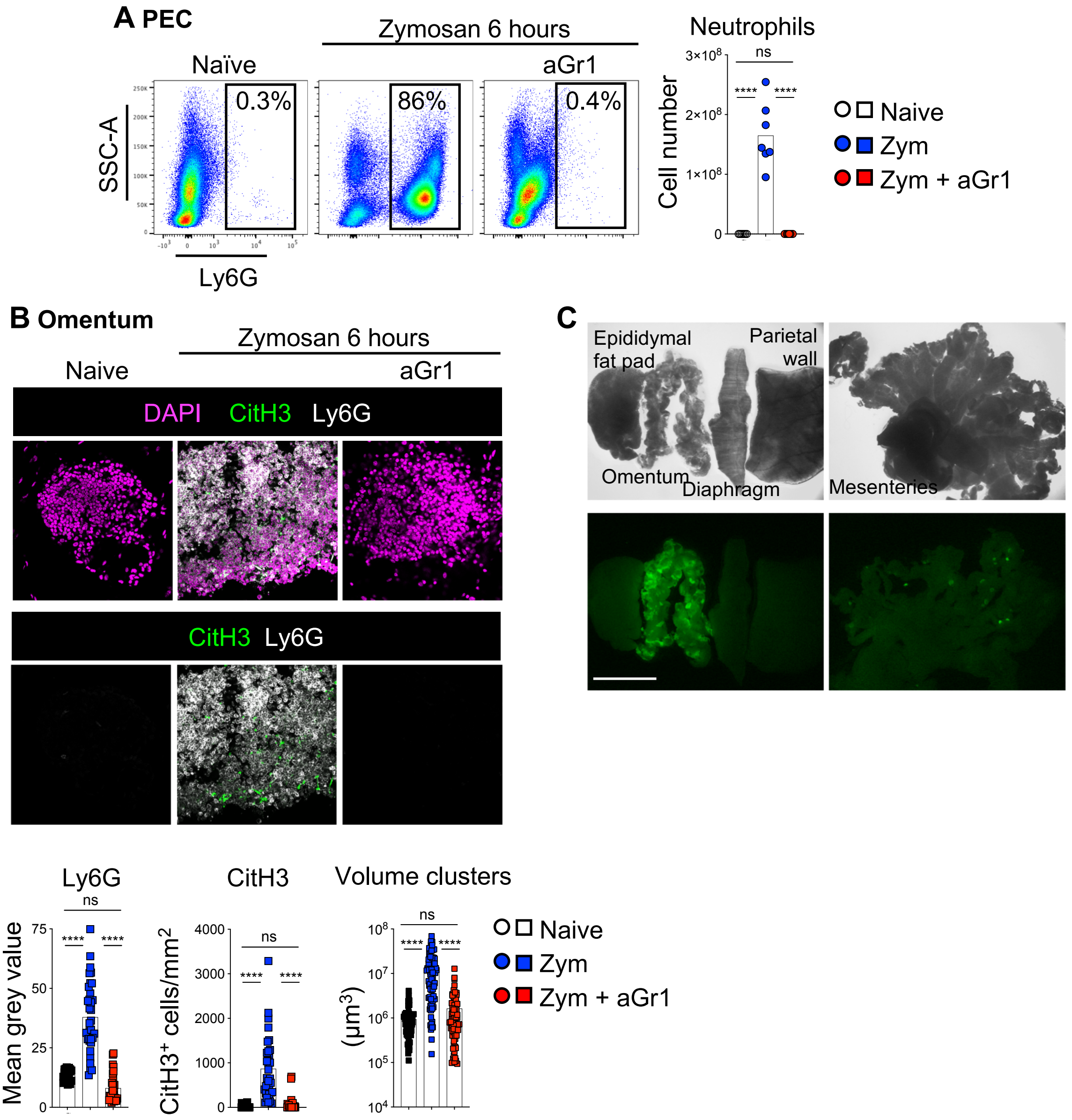

Figure S7. Neutrophil depletion blocks the formation of aggregates recovered with CitH3^{+} DNA and FALCs concentrates Fluo-Zym particles in the omentum and mesenteries related to Figure 4 and 5. **A**, Mice received anti-Gr1 blocking antibodies to deplete neutrophils 24h prior to Zymosan i.p. injection. Flow-cytometric analysis showing frequency of neutrophils in PEC and quantification of the number of neutrophil found in PEC of naïve mice (white circle) , mice 6h post Zymosan-A injection (blue circle) and mice which received anti-Gr1 antibody prior to Zymosan injection (red circle). Data pooled from two independent experiments with n=6 mice per group. **B**, Representative confocal images of wholemount immuno-fluorescence staining of omFALCs from mice as described in **A** with DAPI (magenta), CitH3 (green) and Ly6G (grey); quantification of the mean grey value of Ly6G and the number of CitH3^{+} nuclei in omFALC sections; and quantification of the volume of omFALCs as assessed by measuring the maximal length, width and depth of clusters visualized with DAPI. Data pooled from two independent experiments with n≥25 (mean grey value Ly6G and CitH3^{+} quantification) or n≥50 (cluster size quantification) imaged clusters from n=7 mice per group. **C,** Mice were injected i.p. with Fluo-Zym and the epididymal fat pad, omentum, parietal wall, diaphragm and mesenteries were analyzed 6 hours post injection. Representative stereo-microscope imaging of the tissues showing bright field (top) and Fluo-Zym (bottom). Scale bar 0.5 cm. Data pooled from two independent experiments with n=6 mice per group.

	Pathway	$-Log_{10}(P)$
Matn2⁺ fibroblasts	Elastic fibre formation	1.43891036
	Extracellular matrix organization	1.44523816
	Diseases associated with O-glycosylation of proteins	1.45319981
	Post-translational protein phosphorylation	1.53030527
	Molecules associated with elastic fibres	1.64886348
	IRE1alpha activates chaperones	1.65319331
	O-glycosylation of TSR domain-containing proteins	2.37862719
	Extrinsic Pathway of Fibrin Clot Formation	2.38400989
Ccl11⁺ fibroblasts	Signaling by Interleukins	3.23957752
	Laminin interactions	3.24336389
	Elastic fibre formation	4.17783192
	Collagen formation	4.75945075
	Assembly of collagen fibrils and other multimeric structures	4.7878124
	Integrin cell surface interactions	5.5214335
	Non-integrin membrane-ECM interactions	6.01233374
	Collagen biosynthesis and modifying enzymes	6.03151705
	Regulation of Insulin-like Growth Factor (IGF) transport and uptake by Insulin-like Growth Factor Binding Proteins (IGFBPs)	6.10568394
	Collagen chain trimerization	6.24795155
	Collagen degradation	6.43651891
	Degradation of the extracellular matrix	8.326058
	Axon guidance	8.4034029
	Interleukin-4 and Interleukin-13 signaling	8.82681373
	Extracellular matrix organization	10.4546929
Ifit⁺ mesothelium	Cytokine Signaling in Immune system	1.82320711
	Antiviral mechanism by IFN-stimulated genes	2.31609774
	ISG15 antiviral mechanism	2.57665941
	Regulation of IFNA signaling	2.90374714
	Interferon alpha/beta signaling	7.04000516
	Interferon Signaling	8.65560773
Cxcl13⁺ mesothelium	Interleukin-1 family signaling	2.72327862
	Interferon gamma signaling	2.73810127
	Interleukin-1 signaling	2.79221515
	NIK-->noncanonical NF-kB signaling	2.86431568
	TNFR2 non-canonical NF-kB pathway	2.88167673
	Innate Immune System	3.14327111
	Activation of NF-kappaB in B cells	3.2313619
	Immune System	3.3705904
	Attenuation phase	3.37882372
	Interferon alpha/beta signaling	3.74472749
	Chemokine receptors bind chemokines	3.79317412
	Interferon Signaling	3.95467702
	Signaling by Interleukins	4.84163751
	Cytokine Signaling in Immune system	4.91009489
	Interleukin-10 signaling	6.47366072
Mesothelium	Extracellular matrix organization	2.68782751
	Proton-coupled monocarboxylate transport	2.76531065
	Hemostasis	2.87661033
	Keratan sulfate biosynthesis	3.08196966
	Cell junction organization	3.26042766
	Transport of connexons to the plasma membrane	4.01954211
	Microtubule-dependent trafficking of connexons from Golgi to the plasma membrane	4.1090204
	Cell-Cell communication	4.16941133
	Regulation of Insulin-like Growth Factor (IGF) transport and uptake by Insulin-like Growth Factor Binding Proteins (IGFBPs)	4.34582346
	Formation of tubulin folding intermediates by CCT/TriC	4.51999306
	Gap junction assembly	4.74472749
	Gap junction trafficking and regulation	4.79048499
	Post-chaperonin tubulin folding pathway	4.95860731
	Gap junction trafficking	5.01818139
	Smooth Muscle Contraction	7.81815641

Table S1. Pathway analysis of omentum stromal cell subsets related to Figure 1. Bioreactome analysis of DEGs for each cluster.

	Acute appendicitis	Biliary colic
**Number of participants**	13	10
**Female:Male**	7:6	8:2
**Median age in years (range)**	28 (16 – 58)	48 (22 to 67)
**Body mass index (mean)**	29.1	32.3
**White blood cell count x 10^9/L***	14.78 (5.2 - 19.7)	6.81 (3.90 - 9.80)
**Neutrophil count x 10^9/L***	11.95 (3.16 - 16.81)	4.05 (2.16 - 8.08)
**Lymphocyte count x 10^9/L***	1.63 (0.38 - 2.90)	1.99 (1.13 - 2.74)
**Monocyte count x 10^9/L***	1.10 (0.14 - 2.06)	0.54 (0.35 - 0.75)
**Eosinophil count x 10^9/L***	0.07 (0.01 - 0.06)	0.24 (0.05 - 62.00)
**Basophil count x 10^9/L***	0.02 (0.01 - 0.06)	0.03 (0.01 - 0.05)
**Serum C-reactive protein mg/L**	63 (6 to 215)	4 (< 1 to 5)

*data are mean (range)

Table S2. Summary table of patient demographics related to Figure 7. Sex, Age, body mass index (BMI) white blood cell (WBC) count and C-reactive protein (CRP) characteristics of the Biliary colic patients and acute appendicitis patients recruited for the study.
